# Regulation of *Aspergillus nidulans* CreA-Mediated Catabolite Repression by the F-Box Proteins Fbx23 and Fbx47

**DOI:** 10.1128/mBio.00840-18

**Published:** 2018-06-19

**Authors:** Leandro José de Assis, Mevlut Ulas, Laure Nicolas Annick Ries, Nadia Ali Mohamed El Ramli, Ozlem Sarikaya-Bayram, Gerhard H. Braus, Ozgur Bayram, Gustavo Henrique Goldman

**Affiliations:** a Departamento de Ciências Farmacêuticas, Faculdade de Ciências Farmacêuticas de Ribeirão Prêto, Bloco Q, Universidade de São Paulo, São Paulo, Brazil; b Department of Biology, Maynooth University, Maynooth, Co Kildare, Ireland; c Department of Molecular Microbiology and Genetics, Institute of Microbiology and Genetics, Georg-August-University, Göttingen, Germany; University of Wisconsin-Madison; Yonsei University

**Keywords:** carbon catabolite repression, CreA, F-box, SCF complex, protein kinase

## Abstract

The attachment of one or more ubiquitin molecules by SCF (Skp–Cullin–F-box) complexes to protein substrates targets them for subsequent degradation by the 26S proteasome, allowing the control of numerous cellular processes. Glucose-mediated signaling and subsequent carbon catabolite repression (CCR) are processes relying on the functional regulation of target proteins, ultimately controlling the utilization of this carbon source. In the filamentous fungus Aspergillus nidulans, CCR is mediated by the transcription factor CreA, which modulates the expression of genes encoding biotechnologically relevant enzymes. Although CreA-mediated repression of target genes has been extensively studied, less is known about the regulatory pathways governing CCR and this work aimed at further unravelling these events. The Fbx23 F-box protein was identified as being involved in CCR and the Δ*fbx23* mutant presented impaired xylanase production under repressing (glucose) and derepressing (xylan) conditions. Mass spectrometry showed that Fbx23 is part of an SCF ubiquitin ligase complex that is bridged via the GskA protein kinase to the CreA-SsnF-RcoA repressor complex, resulting in the degradation of the latter under derepressing conditions. Upon the addition of glucose, CreA dissociates from the ubiquitin ligase complex and is transported into the nucleus. Furthermore, casein kinase is important for CreA function during glucose signaling, although the exact role of phosphorylation in CCR remains to be determined. In summary, this study unraveled novel mechanistic details underlying CreA-mediated CCR and provided a solid basis for studying additional factors involved in carbon source utilization which could prove useful for biotechnological applications.

## IMPORTANCE

The production of biofuels from plant biomass has gained interest in recent years as an environmentally friendly alternative to production from petroleum-based energy sources. Filamentous fungi, which naturally thrive on decaying plant matter, are of particular interest for this process due to their ability to secrete enzymes required for the deconstruction of lignocellulosic material. A major drawback in fungal hydrolytic enzyme production is the repression of the corresponding genes in the presence of glucose, a process known as carbon catabolite repression (CCR). This report provides previously unknown mechanistic insights into CCR through elucidating part of the protein-protein interaction regulatory system that governs the CreA transcriptional regulator in the reference organism Aspergillus nidulans in the presence of glucose and the biotechnologically relevant plant polysaccharide xylan.

## INTRODUCTION

In eukaryotic cells, the fate of proteins is regulated by a variety of posttranslational modifications, a process in which specific molecules are attached to target proteins, thereby altering function, activity, or localization (1). Essential cellular functions such as DNA repair, replication, and cell death and differentiation rely on these processes, allowing the temporal control of cellular protein concentrations, which is crucial for developmental processes (2). One of the first posttranslational modifications studied in detail was ubiquitylation, in where a ubiquitin molecule is attached to a substrate protein in a multistep process catalyzed by the sequential action of the E1 ubiquitin-activating enzyme, the E2 ubiquitin-conjugating enzyme, and the E3 ubiquitin ligase (1). The ubiquitin molecule is first bound by E1 in an ATP-dependent process and is subsequently transferred to E2 before E3 covalently ligates the ubiquitin to a lysine residue on the target protein (3–5). The recognition and ubiquitylation of a phosphorylated target protein are catalyzed by the E3 Skip1–Cullin–F-box (SCF) complex, which is composed of the Skp1 adapter protein, the Cullin scaffold protein, and an F-box protein. The cullin subunit binds to Skp1 at the amino terminus, whereas the C terminus binds the Rbx1 RING-finger protein and the E2 enzyme (2). Skp1 interacts with an F-box protein that is responsible for binding to a number of defined protein substrates (1–8) and targeting them for degradation. In addition, some F-box proteins were shown to have an SCF-independent function in fungi and are predicted to form part of centromere-binding, transcription elongation, and translational repressor complexes, as well as being involved in mitochondrial distribution and morphology and the cell cycle (8, 9).

In Saccharomyces cerevisiae, hexose (HXT) transporters, responsible for the uptake of simple sugars such as glucose, are regulated via an SCF complex (10–12). In the absence of glucose, the regulators Mth1p and Std1p and the transcription factor Rgt1p repress the expression of glucose transporter-encoding genes. The presence of extracellular glucose is sensed by Snf3p and Rgt2p, which subsequently activate casein kinase 1/2 (Yck1/2p), resulting in the phosphorylation and nuclear export of Mth1p and Std1p (13–19). Phosphorylated Mth1p and Std1p are recognized by the SCF ubiquitin-protein ligase complex Grr1p, which targets them for proteasome degradation (13, 15, 16, 20), whilst Rgt1p is phosphorylated by protein kinase A, thereby relieving the repression of glucose transporter-encoding genes (14, 16, 21–23).

Glucose uptake and metabolism have gained considerable interest in recent years due to their presenting a drawback in the production of second-generation (2G) biofuels. Second-generation biofuel production relies on the hydrolysis of polysaccharides contained within plant biomass through the combined action of secreted fungal enzymes such as cellulases and xylanases (24). In the presence of glucose, enzyme secretion is inhibited due to carbon catabolite repression (CCR), a mechanism whereby genes encoding hydrolytic enzymes are repressed by the transcription factor CreA, thereby allowing the preferential utilization of the energetically most favorable carbon source (25–29). In contrast to S. cerevisiae, where the regulation of CreA homologue Mig1p has been extensively studied (30–33), much less is known about CreA regulation itself (27). CreA-mediated CCR is dependent on glucose transport and subsequent phosphorylation (34).

The aim of this work was to screen a library of 74 *fbx* deletion mutants and to uncover F-box proteins involved in CCR in Aspergillus nidulans, in order to further unravel the molecular mechanisms and pathways governing glucose utilization in filamentous fungi. Two F-box proteins, Fbx23 and Fbx47, were identified as being important for CCR, with the former working in an SCF ubiquitin ligase complex that is responsible for CreA degradation under carbon catabolite (CC)-derepressing conditions. CreA, which has been shown to form a complex with the corepressors SsnF and RcoA under all tested conditions, translocates to the nucleus in the presence of glucose. Protein kinase GskA was identified as a bridge between the SCF ubiquitin ligase complex and the CreA repressor complex. Furthermore, casein kinase (CkiA) interacts with the SCF complex and is involved in CCR, although the nature of CkiA-mediated phosphorylation remains to be determined. This report provides mechanistic details on the regulation of CreA under CC-repressing and CC-derepressing conditions and further elucidates carbon utilization in the reference organism A. nidulans with the potential to improve fungal biotechnological applications.

## RESULTS

### Identification of the Fbx23 and Fbx47 F-box proteins important for CCR.

**FIG 1:**
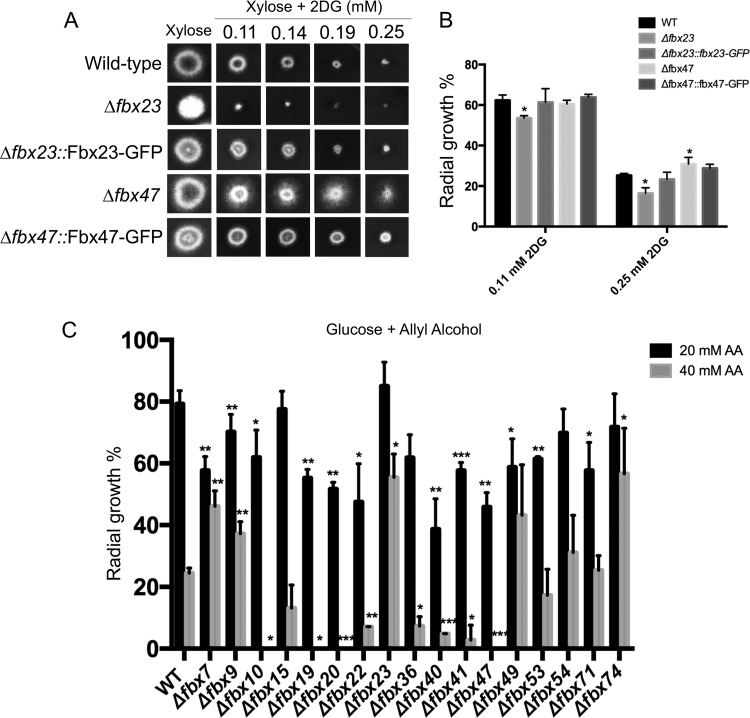
F-box proteins Fbx23 and Fbx47 are involved in CCR. (A) The wild-type, Δ*fbx23*, and Δ*fbx47* strains and respective complemented strains were grown on minimal medium (MM) supplemented with 1% xylose and increasing concentrations of the glucose analogue 2-deoxyglucose (2DG) for 48 h at 37°C. (B) Radial growth diameter after 5 days (percent radial growth compared to control condition without any drugs) at 37°C in two different concentrations of 2DG. (C) Strains were grown on MM supplemented with 1% glucose and increasing concentrations of allyl alcohol before the radial diameters of the strains were measured under all conditions, normalized by the control condition (without allyl alcohol), and statistically compared to the wild-type strain data. Standard deviations are shown for 3 replicates, and statistical differences were calculated using a one-tailed, paired *t* test (*, *P* < 0.05, **, *P* < 0.01; ***, *P* < 0.001).

A total of 74 Fbx (F-box) protein-encoding genes were previously identified in A. nidulans (35), and deletion strains were generated for each gene (see Table S1 in the supplemental material), with the exception of *fbx25* (AN6359), which was shown to be essential, and *fbx50* (AN10516), which was previously deleted and was identified as being required for the sexual development of ascospores (35). The *fbx* deletion collection was subsequently screened for genes involved in CCR by growing all 72 knockout strains in liquid minimal medium supplemented with xylose as a single carbon source and increasing concentrations of the glucose analogue 2-deoxy-glucose (2DG) in the presence of the cellular growth indicator alamarBlue. Although 2DG is phosphorylated by hexokinase during the first step of glycolysis, it cannot be further metabolized and strains presenting increased sensitivity or resistance to this compound have a defect in either CCR-related repression or derepression. Five *fbx* mutant strains were identified, comprising three 2DG-sensitive strains, the *Δfbx20* (AN4535), *Δfbx21* (AN5509), and *Δfbx23* (AN5593) strains, and two 2DG-resistant strains, the *Δfbx47* (AN8909) and *Δfbx55* (AN5941) strains (not shown). When grown on solid medium supplemented with different concentrations of 2DG, only the *Δfbx23* and *Δfbx47* strains were confirmed as being highly sensitive and resistant to 2DG, respectively, compared to the wild-type (WT) strain (Fig. 1A). There was reduced radial growth and conidiation in the *Δfbx23* mutant compared with the corresponding wild-type strain (Fig. 1A). Complementation of the corresponding strains with the *gfp*-tagged gene resulted in growth phenotypes similar to the wild-type phenotypes, indicating that the 2DG sensitivity/resistance was due to the lack of *fbx23* and *fbx47* (Fig. 1A and B).

10.1128/mBio.00840-18.6 TABLE S1  A. nidulans strains used in this study. Download TABLE S1, DOCX file, 0.1 MB. Copyright © 2018 de Assis et al. 2018 de Assis et al. This content is distributed under the terms of the Creative Commons Attribution 4.0 International license.

It has been shown that increased resistance or sensitivity to 2DG can also be due to a reduction in glucose uptake in a mutation-dependent manner (30, 31, 36). Subsequently, a second screen was carried out, in order to further determine CCR-related defects in the *fbx* deletion collection. Strains were grown on minimal medium supplemented with glucose and increased concentrations of allyl alcohol (AA), an alcohol analogue that is converted by alcohol dehydrogenase to the toxic compound acrolein (32). The CCR-derepressed strains were more sensitive to this analogue than the CCR-repressed strains. A total of 18 *fbx* deletion strains (Fig. 1C), including the Δ*fbx23* and Δ*fbx47* mutants, were identified as having increased resistance or sensitivity to AA compared with the wild-type strain, with the Δ*fbx23* and Δ*fbx47* mutants being resistant and sensitive, respectively (Fig. 1C). Based on these initial screens, the *Δfbx23* and *Δfbx47* strains were chosen for further analysis as they presented phenotypes in both 2DG and AA screens, suggesting that they are involved in CCR, and as they also have previously not been characterized.

### Xylanase gene expression and secretion are deregulated in the *Δfbx23* and *Δfbx47* strains.

**FIG 2:**
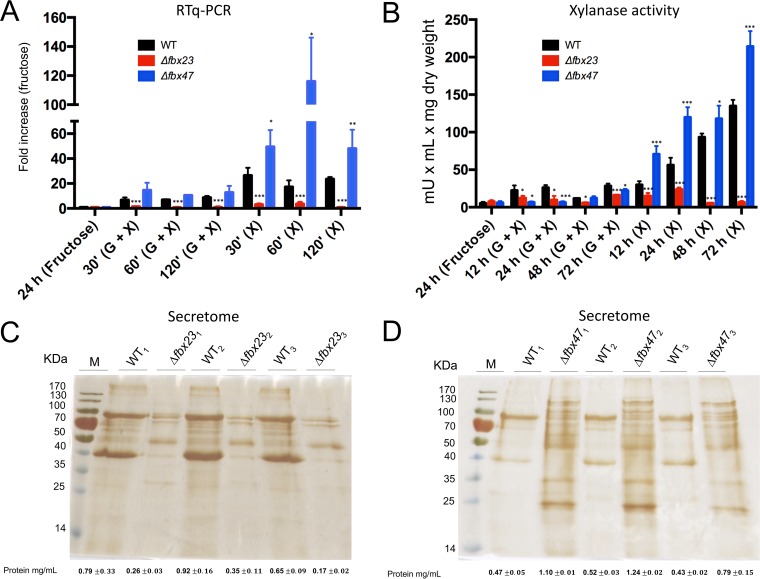
Fbx23 and Fbx47 are involved in the secretion of extracellular proteins. (A) Expression of *xlnA* in the WT (wild-type), Δ*fbx23*, and Δ*fbx47* strains when grown in MM (minimal medium) supplemented with 1% fructose for 24 h (control) and after transfer to MM supplemented with 1% xylose [derepressing; (X)] or 1% xylose plus 2% glucose [repressing; (G + X)] for 30, 60, and 120 min. (B) Extracellular xylanase activity under the same conditions as those described for panel A, although at later time points (12, 24, 48, and 72 h). (C and D) Silver-stained gel of secreted proteins of 3 biological replicates in the WT strain and Δ*fbx23* (C) and Δ*fbx47* (D) strains after transfer from fructose-rich to xylose-rich MM for 72 h. Also shown is the total protein concentration for each sample as measured by Bradford assay. Standard deviations represent the average results from 3 biological replicates; statistical differences were calculated using a paired *t* test (*, *P* < 0.05, **, *P* < 0.01; ***, *P* < 0.001) based on comparison with the WT strain.

A known target of CreA-mediated CCR is endo-1,4-β-xylanase-encoding gene *xlnA* (25, 26, 33). To determine a potential effect of the observed CCR-related phenotypes on xylanase expression in the *Δfbx23* and *Δfbx47* strains, the expression of *xlnA* was assessed by quantitative reverse transcription real-time PCR (RT-qPCR) when strains were grown for 24 h in minimal medium supplemented with fructose (a noninducing condition) and after transfer to xylose (1% [wt/vol])-rich minimal media (inducing condition) or to minimal media supplemented with both xylose (1% [wt/vol]) and glucose (2% [wt/vol], repressing condition) for 30, 60, and 120 min. In the Δ*fbx23* strain, *xlnA* expression was significantly reduced compared to the wild-type strain expression under all tested conditions (Fig. 2A). On the other hand, *xlnA* expression was significantly increased in the presence of xylose in the Δ*fbx47* strain compared to the wild-type strain (Fig. 2A), whereas no difference in the level of *xlnA* expression was observed in the Δ*fbx47* strain in the simultaneous presence of xylose and glucose compared to the wild-type strain. These results suggest that Fbx23 is required for *xlnA* induction under all conditions, whereas the deletion of *fbx47* significantly increased *xlnA* expression under inducing conditions but not under repressing conditions.

To confirm if the observed gene expression patterns were accompanied by similar protein secretion profiles, xylanase activity analysis and characterization of total secreted proteins were performed when strains were grown for 24 h in fructose minimal medium (MM) and transferred to MM supplemented with 1% (wt/vol) xylose as a single carbon source (inducing condition) or 1% (wt/vol) xylose and 2% (wt/vol) glucose (repressing condition) for 1, 2, and 3 days. Both the Δ*fbx23* and Δ*fbx47* strains showed a slight reduction in xylanase activity in the simultaneous presence of xylose and glucose compared to the wild-type strains, whereas xylanase activity in the wild-type strain and both mutant strains after transfer from fructose to xylose plus glucose remained low due to CCR (Fig. 2B). As expected, transfer to xylose-rich media triggered a significant increase in xylanase secretion in the wild-type strain, which was not observed in the Δ*fbx23* strain (Fig. 2B). Furthermore, the Δ*fbx47* strain had significantly increased extracellular xylanase activity under inducing conditions compared to the wild-type strain (Fig. 2B). In addition, total secreted protein quantification and profiling suggested a severe reduction in protein secretion in the Δ*fbx23* strain and a significant increase in protein secretion in the Δ*fbx47* strain (Fig. 2C and D). These results are in agreement with the gene expression analysis and indicate that deletion of *fbx23* had a repressive effect on xylanase gene expression and secretion under all tested conditions, whereas deletion of *fbx47* resulted in a hypersecretion phenotype under inducing conditions. These results therefore suggest that Fbx23 and Fbx47 are involved in xylanase induction or in CCR or both and ultimately influence xylanase production and secretion.

### Identification of Fbx23 and CreA protein interaction partners.

To further understand the regulatory influence of Fbx23 and Fbx47 in glucose utilization and CCR, protein interaction studies using TAP (tandem affinity purification)-tagged Fbx23, Fbx47, and CreA proteins were carried out. Functional strains (see Fig. S1 in the supplemental material), which actively expressed the tagged Fbx23 and CreA proteins, were successfully constructed, whereas expression of TAP-tagged (or green fluorescent protein [GFP]-tagged) Fbx47 could not be detected by Western blotting in the respective strains when grown under either glucose-rich or xylose-rich conditions. Fusion of the TAP tag after another 2 predicted stop codons (F1 and F2) in the C terminus of Fbx47 also did not result in strains expressing the tagged protein, which could not be detected using mass spectrometry (MS) analysis (Fig. S2). We did not try to add the TAP tag at the N terminus of Fbx47 as this could potentially influence the function of the protein because of the presence of the F-box domain in this region.

10.1128/mBio.00840-18.1 FIG S1  Protein-tagged strains are functional. Strains were grown (point inoculation) on minimal medium supplemented with glucose at 37°C for 48 h. Strains were constructed in either the AGB551 background (CreA::TAP, Fbx23::TAP, CreA::GFP, Fbx23::GFP, Fbx23::3×HA, GskA::GFP, GskA::3×HA, CreA::GFP GskA::3×HA, Fbx23::3×HA GskA::GFP, and Fbx23::GFP GskA::3×HA) or the TNO2a3 background (CreA::FLAG and GskA::GFP CreA::FLAG). The Fbx23::3×HA GskA::GFP strain has a sick-growth phenotype. Download FIG S1, PDF file, 1.1 MB. Copyright © 2018 de Assis et al. 2018 de Assis et al. This content is distributed under the terms of the Creative Commons Attribution 4.0 International license.

10.1128/mBio.00840-18.2 FIG S2  Fbx47 could not be tagged at the C terminus and detected. (A) Diagram showing the size of the full-length TAP-tagged Fbx47 protein and of two variants (F1 and F2) with predicted stop codons at different sites. (B) Western blot of protein extracts from the wild-type (WT), full-length, F1, and F2 TAP-tagged strains when grown under glucose-rich or xylose-rich conditions (the blue arrow indicates the predicted position of the full-length Fbx47 protein). The protein could not be detected after IP (immunoprecipitation) by mass spectrometry under the same conditions as those described for panel B. Download FIG S2, PDF file, 0.3 MB. Copyright © 2018 de Assis et al. 2018 de Assis et al. This content is distributed under the terms of the Creative Commons Attribution 4.0 International license.

**FIG 3:**
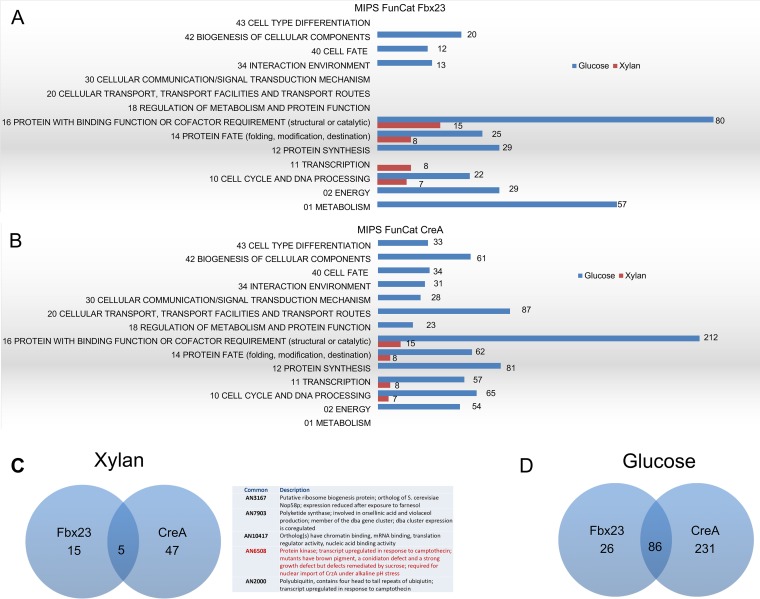
Fbx23 and CreA potentially interact with proteins involved in different cellular processes in the presence of glucose. (A and B) MIPS FunCat categorization of the proteins, identified by mass spectrometry (MS), interacting with Fbx23-TAP (A) and CreA::TAP (B) in the presence of xylan (red) or after the addition of glucose (blue). Due to overlap of functions, one protein can be classified in two or more categories. The exact number of proteins identified in each category is also shown. (C and D) Venn diagrams under conditions of xylan treatment (the table shows proteins that were identified as interacting with both CreA and Fbx23; in red, GskA) (C) and glucose treatment (D).

We therefore decided to proceed with the Fbx23::TAP and CreA::TAP immunoprecipitations (IP) and subsequent MS analysis of the respective potential interacting proteins (a strain expressing nontagged Fbx23 and CreA was used as a negative control, and unspecific interactions were subtracted from the replicates). All strains were grown in minimal medium supplemented with 1% (wt/vol) xylan (inducing condition) for 24 h before glucose (repressing condition) was added to reach a final concentration of 2% (wt/vol) for 5, 10, 15, and 30 min. All putative Fbx23-interacting and CreA-interacting proteins were categorized according to MIPS FunCat (Munich Information Center for Protein Sequences Functional Categorization) (Fig. 3). In the presence of xylan, Fbx23 potentially interacted with proteins having functions in the cell cycle and protein fate and binding, whereas addition of glucose showed enrichment in proteins involved in metabolism, energy, protein synthesis, interaction with the environment, cell fate, and biogenesis (Fig. 3A). Similarly, in the presence of xylan, MIPS FunCat categorization showed potential interactions of CreA::TAP with proteins involved in cell cycle, transcription, and protein fate and binding, whereas the addition of glucose showed enrichment of proteins with binding function and of proteins involved in cellular transport and protein synthesis as well as proteins having functions in diverse cellular processes such as energy, cell cycle, transcription, protein fate, metabolism, cellular communication, interaction with the environment, cell fate, biogenesis of cellular components, and cell type differentiation (Fig. 3B). In the presence of xylan, Fbx23 had 15 and CreA 47 unique interactions, whereas both interacted with the same 5 proteins, including protein kinase GskA and polyubiquitin precursor Ubi4 (Fig. 3C). Under repressing conditions, Fbx23 had 26 and CreA had 231 unique interactions, as well as having 85 common interactions (Fig. 3D). These results suggest that both Fbx23 and CreA play major roles during glucose utilization and that CreA seems to be involved in several cellular processes other than CCR.

### In-depth analysis of Fbx23 and CreA interacting partners.

Next, the profiles of the identified Fbx23 and CreA interacting proteins were further analyzed. In the presence of xylan, SCF complex proteins SkpA, CulA, and NeddH as well as the E3 ligase RbxA were coimmunoprecipitated (Co-IP) with Fbx23, suggesting that Fbx23 is part of an SCF ubiquitin ligase complex (see Data Set S1 in the supplemental material). Upon the addition of glucose, the same proteins were identified, except for RbxA, suggesting loosening and/or inactivation of the SCF complex. Furthermore, protein kinases GskA and CkiA also coimmunoprecipitated with Fbx23 in the presence of xylan and up to 15 min after glucose addition (Data Set S1). These results suggest that Fbx23 is part of the SCF complex in the presence of alternative carbon sources, whereas addition of glucose causes the dissociation of E3 ligase RbxA, thereby promoting the inactivation of the SCF complex. Furthermore, Fbx23 interacted with protein kinases GskA and CkiA under xylan-rich conditions and this interaction was lost after 30 min in glucose-rich medium, suggesting that these kinases may be involved in phosphorylating potential SCF targets under CCR-derepressing conditions.

10.1128/mBio.00840-18.8 DATA SET S1  Fbx23-TAP-interacting proteins, as identified by LC-MS/MS (two replicates), and subtracted unspecific interactions from the control (Wild-type) under the same experimental conditions. Download DATA SET S1, XLSX file, 1 MB. Copyright © 2018 de Assis et al. 2018 de Assis et al. This content is distributed under the terms of the Creative Commons Attribution 4.0 International license.

Analysis of the CreA::TAP immunoprecipitated proteins in the presence of xylan also showed putative interaction of CreA with protein kinase GskA, suggesting that the latter forms a bridge between CreA and the Fbx23 SCF ubiquitin ligase complex. Furthermore, CreA interacted with SsnF and RcoA under all tested conditions. SsnF and RcoA are the homologues of S. cerevisiae Ssn6p and Tup1p, which, together with Mig1p, form a transcriptional repressor complex (37). In addition, the polyubiquitin precursor molecule Ubi4 was also identified as interacting with CreA complex in the presence of xylan but disappeared within 10 min of incubation in glucose-rich medium. This confirms previous observations that (de)ubiquitylation plays a role in controlling the amount of CreA present within the cell under CCR derepressing and repressing conditions (27, 38).

CreA moves to the nucleus upon the addition of glucose (27, 39), and potential CreA interaction partners included the karyopherins/importins KapB and KapI. Karyopherins are a class of nuclear import/export proteins that control the movement of molecules between the nucleus and the cytoplasm (34, 40). In S. cerevisiae, cytoplasmic-nuclear shuttling of Mig1p is also regulated by importins (41–43). It is possible that the essential importin KapB may be responsible for and/or involved in CreA nuclear transport. Furthermore, the addition of glucose promoted interaction of CreA with Sin3, a component of a histone deacetylase (HDAC) complex which is responsible for the deacetylation of lysine residues in the N-terminal region of histones (44) (Data Set S2). In S. cerevisiae, in the presence of high concentrations of glucose, *SUC2* expression is repressed by the Ssn6-Tup1 complex which is thought to directly interact with histone deacetylases (HDACs), thereby promoting hypoacetylation of histones H3 and H4 and ensuring gene repression (37, 45, 46). It is possible that the recruitment of Sin3 to the RcoA-SsnF-CreA complex in A. nidulans in the presence of glucose assumes a function similar to the one described in yeast.

10.1128/mBio.00840-18.9 DATA SET S2  CreA-TAP-interacting proteins, as identified by LC-MS/MS (two replicates), and subtracted unspecific interactions from the control (Wild-type) under the same experimental condition. Download DATA SET S2, XLSX file, 1 MB. Copyright © 2018 de Assis et al. 2018 de Assis et al. This content is distributed under the terms of the Creative Commons Attribution 4.0 International license.

### Fbx23 is localized in the cytoplasm and is required for CreA::GFP degradation under CC-derepressing conditions.

**FIG 4:**
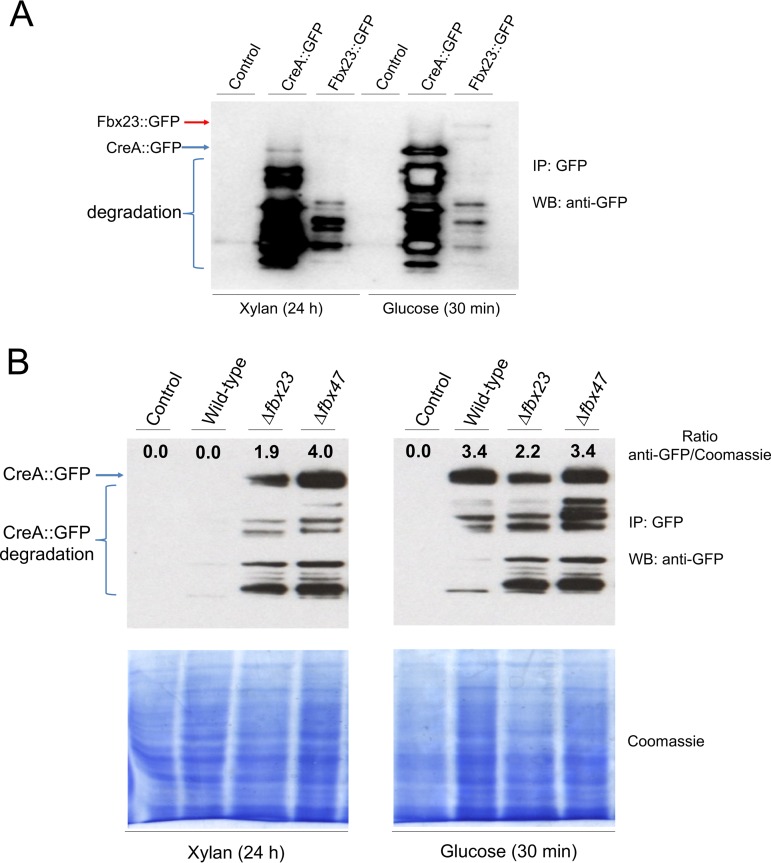
Stability of CreA under derepressing and repressing conditions. (A) Western blot of immunoprecipitated (IP) CreA::GFP and Fbx23::GFP when grown in carbon catabolite (CC) under derepressing (xylan) or repressing (glucose) conditions. Full-length CreA::GFP (76 kDa) is indicated by a blue arrow, whereas full-length Fbx23::GFP (112 kDa) is indicated by a red arrow. WB, Western blot. (B) Western blot of IP CreA::GFP in different background strains when grown under the conditions specified for panel A (full-length CreA::GFP is indicated by a blue arrow). The amount of CreA::GFP (anti-GFP detection/Coomassie blue staining ratio) for each strain after normalization by Coomassie-stained total protein content is also shown. CreA::GFP (76-kDa) degradation products are indicated.

Next, the potential protein interactions identified by MS as described above were validated. The presence and stability of the GFP-tagged CreA and Fbx23 proteins were assessed by Western blotting. In agreement with reference 27, a faint band of CreA could be detected in the presence of xylan, which became much stronger upon the addition of glucose for 30 min, suggesting an increase in the amount of cellular CreA (Fig. 4A). Similarly, Fbx23::GFP was detected only under glucose-rich conditions and not in the presence of xylan (Fig. 4A). These results indicate degradation of CreA and Fbx23 in the presence of CC-derepressing conditions and an increase in cellular pools of these two proteins in the presence of glucose.

Next, the presence and stability of CreA::GFP were verified by Western blotting in the wild-type (WT) and *Δfbx23* and *Δfbx47* background strains when grown under the conditions specified above. In agreement with Fig. 4A, the amount of CreA increased in the presence of glucose compared to xylan in the WT strain, whereas no CreA was detected in the negative control (non-GFP-tagged) strain (Fig. 4B). CreA abundance was determined by normalizing the amount of CreA by the total protein input (Fig. 4B). Deletion of *fbx23* and *fbx47* resulted in accumulation of the CreA protein in the presence of xylan but had no effect on the amount of CreA under glucose-rich conditions compared to the levels seen with the wild-type strain (Fig. 4B). These results suggest that Fbx23 (as well as Fbx47) is required for the destabilization of CreA under derepressing conditions, confirming the hypothesis that the E3 ligase SCF complex promotes CreA degradation.

The accumulation of CreA in the Δ*gskA* strain could not be determined due to its strong growth and conidiation defects (47), which prevented the mycelial biomass accumulation required for immunoprecipitation and subsequent Western blotting.

To further confirm the Western blot results regarding the presence and stability of CreA::GFP in the different deletion strains, microscopy was carried out when strains were grown overnight in minimal medium supplemented with xylan or glucose. CreA::GFP in the wild-type strain was mostly localized in the nucleus in the presence of glucose, whereas the growth in xylan caused relocalization to the cytoplasm (Table 1). In the *Δfbx23* strain, however, CreA::GFP was observed in the nuclei under both derepressing and repressing conditions. These results suggest that Fbx23 is required for the degradation or the nuclear translocation of CreA or both in the absence of glucose. CreA::GFP nuclear localization was reduced in glucose and increased in xylan in the *Δfbx47* strain compared to the wild-type strain, suggesting that this protein is also required for correct CreA cellular localization (Table 1). The cellular localization of Fbx23::GFP was also assessed and found to be cytoplasmic under repressing and derepressing conditions (Table 1). Fluorescence of Fbx23::GFP was absent under CCR-derepressing conditions, confirming the degradation under those conditions, whereas the intensity of Fbx23::GFP fluorescence increased upon the addition of glucose (Fig. S4). These results suggest that the predicted E3 ligase SCF complex-mediated ubiquitylation predominantly takes place in the cytoplasm under the conditions specified here and confirm that the Fbx23 protein complex is degraded in the presence of xylan (Fig. 4A; see also Fig. S4).

**TABLE 1:** Percentages of CreA::GFP, Fbx23::GFP, CkiA::GFP, and GskA::GFP nuclear localization^a^

Background	% nuclear localization	
	Glucose	Xylan
CreA::GFP		
A. nidulans wild type	95.61	14.02
*A. nidulans Δfbx23*	92.31	94.93
*A. nidulans Δfbx47*	63.79	52.58
	
GFP		
A. nidulans Fbx23::GFP	0.00	0.00
A. nidulans CkiA::GFP	100.00	81.58
A. nidulans GskA::GFP	8.00	45.25

a Strains were grown for 16 h at 22°C in minimal medium supplemented with either glucose or xylan before GFP cellular localization was assessed. Nuclei were counted for at least 100 hyphal germlings under each condition and subjected to Hoechst 33258 staining in order to confirm GFP colocalization.

### Casein kinase is required for CCR regulation.

**FIG 5:**
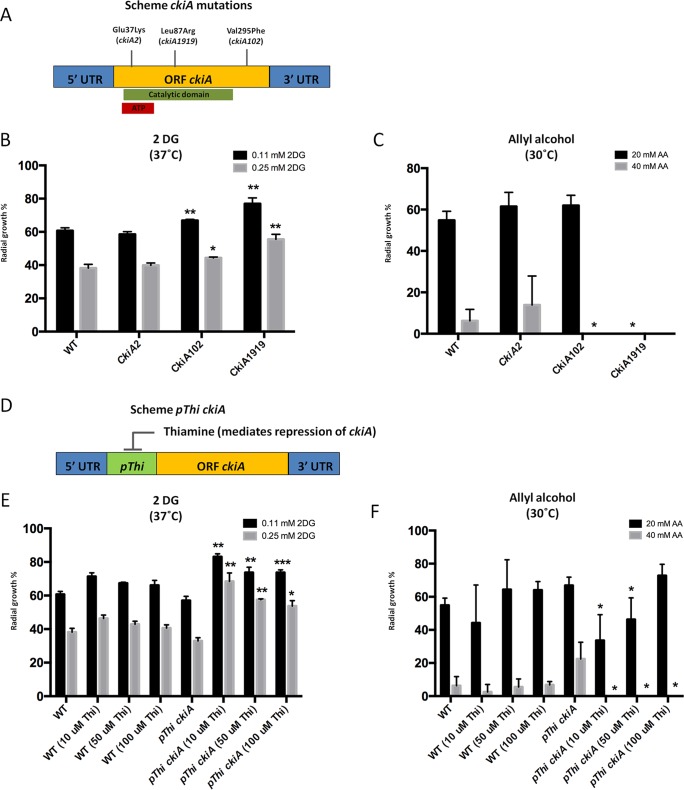
CkiA is required for CCR. (A) Diagram depicting locations of *ckiA* gene point mutations and corresponding amino acid replacements. The green bar shows the catalytic domain of casein kinase and the red bar the ATP binding domain. Strain names are indicated in brackets (Glu, glutamine; Lys, lysine; Leu, leucine; Arg, arginine; Val, valine; Phe, phenylalanine). ORF, open reading frame; UTR, untranscribed region. (B and E) Radial diameters of strains grown for 5 days at 37°C on minimal medium (MM) supplemented with xylose and increasing concentrations of 2-deoxyglucose (2DG). (C and F) Radial diameters of strains grown for 5 days at 30°C on MM supplemented with glucose and increasing concentrations of allyl alcohol (AA). (D) Diagram depicting the conditional mutant where *ckiA* was placed under the control of the thiamine repressible promoter (pThi). Thiamine was added in increasing concentrations to the strains represented in panels E and F to ensure *ckiA* gene repression. The percent radial diameter data show reductions in growth compared to the control condition (without 2DG or AA). Standard deviations are shown for 3 replicates, and statistical differences were calculated using a one-tailed, paired *t* test (*, *P* < 0.05, **, *P* < 0.01; ***, *P* < 0.001) based on the comparison of the mutant strains to the wild-type strain.

In addition to protein kinase GskA, the casein kinase, CkiA, was identified during mass spectrometry as interacting with Fbx23. As CkiA is encoded by an essential gene (48), we used three strains carrying single point mutations in *ckiA* (Fig. 5A) as well as a conditional mutant, where *ckiA* was placed under the control of the thiamine-repressible promoter *thiA* (Fig. 5D), for further analysis and characterization. The *ckiA2* strain contains a glutamine 37-to-lysine substitution in the ATP binding site domain; the *ckiA1919* strain has a leucine 87-to-arginine substitution in the protein kinase catalytic domain; and strain *ckiA102* contains a valine 295-to-phenylalanine substitution (48) (Fig. 5A). All strains were grown in the presence of increasing concentrations of 2DG and AA as described above before colony growth was measured. Both the *ckiA102* and *ckiA1919* mutants were resistant to 2DG and sensitive to AA compared to the wild-type strain (Fig. 5B and C). Further validation of the result described above was shown using a conditional CkiA mutant (Fig. 5D); to characterize the effect of *ckiA* repression on CCR, increased concentrations of thiamine were added in the control strain also. The results showed no effect on the radial diameter experiments that were carried out; however, the conditional mutant had strong resistance to 2DG, especially using a higher concentration (0.25 mM) than the wild-type strain, and the conditional mutant, on the other hand, had sensitivity to allyl alcohol (Fig. 5E and F). The aforementioned results indicate that CkiA is potentially involved in CCR.

To provide additional evidence for the involvement of CkiA in CCR, microscopy of the CkiA::GFP strain was performed under CC-derepressing (xylan) and -repressing (glucose) conditions in order to assess its cellular localization. CkiA::GFP was observed in the cytoplasm and in the nucleus under CC-repressing and CC-derepressing conditions, with apparent stronger GFP fluorescence in the cytoplasm in the presence of glucose, whereas incubation in the presence of xylan resulted in increased GFP fluorescence in the nucleus (Table 1; see also Fig. S3). These results are in agreement with those reported in reference 48 and with the MS data (Data Set S2) and suggest that CkiA colocalizes with Fbx23 in the cytoplasm in the presence of CC-derepressing conditions, whereas the addition of glucose caused relocalization to the nucleus and potential dissociation from the Fbx23 complex.

10.1128/mBio.00840-18.3 FIG S3  Cellular localization of Fbx23, CkiA, and GskA. GFP-tagged strains were grown in minimal medium supplemented with xylan or glucose for 16 h at 22°C, with the exception of GskA::GFP, for which glucose was added to the xylan-grown hyphae for 15 min. Nuclei were subjected to Hoechst staining at room temperature for 5 min. (DIC, differential interference contrast). White arrows indicate the absence of GskA::GFP in the nucleus. Download FIG S3, PDF file, 1.7 MB. Copyright © 2018 de Assis et al. 2018 de Assis et al. This content is distributed under the terms of the Creative Commons Attribution 4.0 International license.

### GskA has a weak interaction with CreA and SCF ubiquitin ligase complex.

To further validate a potential interaction of protein kinase GskA with the Fbx23 SCF ubiquitin ligase and/or CreA repressor complexes, microscopy of the GskA::GFP was first carried out in order to assess cellular localization. GskA::GFP was observed in both the cytoplasm and nucleus in the presence of xylan, but upon the addition of glucose, it became predominantly cytoplasmic (Table 1; see also Fig. S3). These results are in agreement with the MS data, which suggested that GskA is present in the cytoplasm under CC-derepressing conditions and that, upon the addition of glucose, it dissociates from the CreA repressor complex and leaves the nucleus.

In order to confirm a potential physical interaction between Fbx23, GskA, and CreA, immunoprecipitations (IP) of GskA was carried out using two different protein tags (3× hemagglutinin [3×HA] and GFP). GskA::3×HA was first immunoprecipitated, and samples were then run on a gel and transferred to a membrane before being probed for CreA::GFP and Fbx23::GFP with an anti-GFP antibody. No CreA/Fbx23::GFP was detected under derepressing and repressing conditions (Fig. S4). The absence of detectable protein may have been due to the amount of protein present during the interaction under the conditions using xylan-rich medium, where they are predicted to be degraded (Fig. 4A; see also Fig. S5B), and/or due to a weak interaction that can be detected only by MS (Data Sets S1 and S2). We also tried to use IP for analysis of CreA::GFP/FLAG and Fbx23::GFP/3×HA first before probing for GskA with an anti-HA or anti-GFP antibody, but the GskA::3×HA and GskA::GFP tags interacted with the IP resin in control conditions, thereby devalidating the assay (Fig. S4 and S5).

10.1128/mBio.00840-18.4 FIG S4  Interaction of GskA::3×HA with CreA::GFP and Fbx23::GFP by Western blotting. Strains were grown in minimal medium supplemented with xylan for 24 h (A) before glucose was added to reach a final concentration of 2% (wt/vol) for 30 min. (B). The red arrow indicates full-length Fbx23 (112 kDa), and the blue arrow indicates the position of full-length CreA (76 kDa). HA-tagged protein was immunoprecipitated (IP) first, run on a gel, and blotted before membranes were probed with the anti-GFP antibody. Download FIG S4, PDF file, 0.2 MB. Copyright © 2018 de Assis et al. 2018 de Assis et al. This content is distributed under the terms of the Creative Commons Attribution 4.0 International license.

10.1128/mBio.00840-18.5 FIG S5  Interaction of GskA with CreA::GFP and Fbx23::GFP by Western blotting. Strains were grown in minimal medium supplemented with xylan for 24 h before glucose was added to reach a final concentration of 2% (wt/vol) for 30 min. The red arrow indicates full-length Fbx23 (112 kDa), and the blue arrow indicates the position of full-length CreA (76 kDa). (A) CreA::GFP was immunoprecipitated (IP) first, run on a gel, and blotted before membranes were probed for GskA::3×HA with anti-HA antibody. (B) GFP-tagged protein was immunoprecipitated (IP) first, run on a gel, and blotted before membranes were probed with the anti-GFP antibody. (C) CreA::FLAG was immunoprecipitated (IP) first, run on a gel, and blotted before membranes were probed for GskA::GFP with anti-GFP antibody. Download FIG S5, PDF file, 0.6 MB. Copyright © 2018 de Assis et al. 2018 de Assis et al. This content is distributed under the terms of the Creative Commons Attribution 4.0 International license.

Furthermore, mass spectrometry (MS) of GskA::3×HA was also carried out under conditions of growth in the presence of xylan and after addition of 2% (wt/vol) glucose for 30 min (the same conditions as described above). Under CC-derepressing conditions, GskA::3×HA potentially interacts with RcoA (CreA partner) and CkiA (Fbx23 partner) (Data Set S3). Addition of glucose to the cultures resulted in a loss of these interactions. These data suggest the formation of an Fbx23-CkiA-GskA-CreA complex under CC-derepressing conditions (Data Set S3). We were unable to identify CreA or Fbx23 directly in the GskA MS protein data, suggesting a weak/transient interaction between these proteins. Moreover, the MS data showed potential interactions of GskA with the karyopherins KapA, KapB, and KapJ under CC-derepressing conditions and with KapA and KapB under CCR conditions (Data Set S3), suggesting that this protein kinase may be involved in the nuclear/cytoplasmic translocation of CreA and/or CkiA. Furthermore, GskA was identified as potentially interacting with a putative 26S proteasome regulatory subunit (AN1700) (Data Set S3) under CC-derepressing conditions, indicating a function and/or participation in CreA degradation under these conditions. The nature of the mechanism underlying GskA-mediated phosphorylation is under investigation.

10.1128/mBio.00840-18.10 DATA SET S3  GskA-3×HA-interacting proteins, as identified by LC-MS/MS (two replicates), and subtracted unspecific interactions from the control (wild-type) under the same experimental condition. Download DATA SET S3, XLSX file, 0.6 MB. Copyright © 2018 de Assis et al. 2018 de Assis et al. This content is distributed under the terms of the Creative Commons Attribution 4.0 International license.

### RcoA interacts with CreA under all conditions, but *rcoA* is not important for xylanase gene induction.

**FIG 6:**
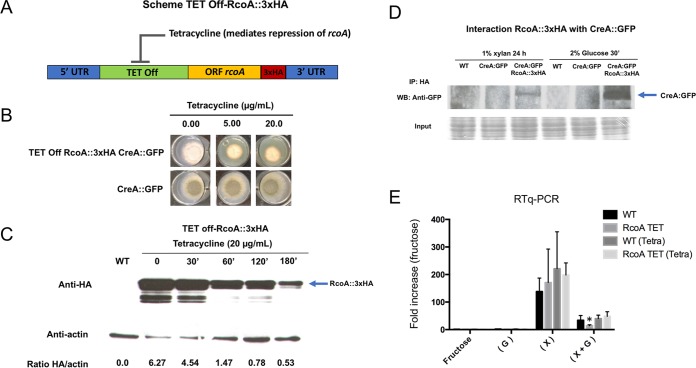
RcoA physically interacts with CreA but is not required for CCR. (A) Diagram depicting the conditional mutant where *rcoA* is controlled by the TET off promoter and tagged with 3×HA in the CreA::GFP background strain. (B) Growth phenotypes of TET off-RcoA::3×HA CreA::GFP and CreA::GFP grown in MM without and with different tetracycline concentrations for 72 h at 37°C. (C) Western blot of TET off-RcoA::3×HA when grown in minimal medium supplemented with glucose for 24 h and after the addition of tetracycline (20 µg/ml) for 30, 60, 120, and 180 min. TET off-RcoA::3×HA was detected with anti-HA antibody (blue arrow), and protein content was normalized by sample actin content (HA/actin ratio). (D) Western blot of TET off-RcoA::3×HA CreA::GFP strain after immunoprecipitation (IP) of HA-tagged protein and incubation with anti-GFP antibody. Strains were grown for 24 h under xylan-rich conditions before glucose was added for 30 min. The wild-type (WT) and CreA::GFP strains were IP controls, whereas Coomassie blue straining of total protein before IP is indicated as the loading control. (E) RTq-PCR showing the expression of *xlnA* in the wild-type (WT) and TET off-RcoA::3×HA CreA::GFP strains when grown in MM supplemented with fructose (control, noninducing) for 24 h and after transfer to glucose-rich (G), xylose-rich (X), or glucose-rich and xylose-rich (X + G) conditions for 1 h. To ensure *rcoA* repression, 20 µg/ml tetracycline was added to the control cultures for a minimum of 3 h and after transfer to the different carbon sources.

The mass spectrometry (MS) data showed that CreA interacted with RcoA and SsnF (encoded by an essential gene), under all conditions tested here. In S. cerevisiae, Mig1p, the homologue of CreA, interacts with the corepressors Ssn6p (SsnF homologue) and Tup1p (RcoA homologue), which are crucial for ensuring Mig1p repressor function (49–51). Deletion of A. nidulans *rcoA* has previously been shown to not result in CC derepression (50) but affects the chromatin structure of target promoters (52–54) and is involved in *veA*-dependent sexual development (55). A direct physical interaction between CreA and RcoA has so far not been shown in A. nidulans. Deletion of *rcoA* results in a strain with a severely reduced growth phenotype (53, 55), and to further validate the MS data and to address the potential involvement of RcoA in CCR, we constructed a conditional mutant, where the *rcoA* expression was regulated by the *tet off* promoter (tetracycline-repressible promoter) and tagged with 3×HA in the background of CreA::GFP (Fig. 6A). The TET off-RcoA::3×HA strain showed 80% of the wild-type radial growth in the absence of tetracycline, whereas the addition of tetracycline severely reduced growth, suggesting that the *rcoA* gene was repressed (Fig. 6B). It is possible that the observed reduction in growth in the absence of tetracycline was due to lower *rcoA* levels in the TET off::RcoA::3×HA strain than in the wild-type strain, and this could have an effect on TET off-RcoA::3×HA growth. Nevertheless, as the main objective was to repress *rcoA* expression, this strain was used here in additional experiments. To confirm the successful repression of *rcoA*, RcoA protein levels were assessed by Western blotting and anti-HA antibody detection after the addition of 20 µg/ml tetracycline treatment for different amounts of time. After 180 min, there was a clear reduction in RcoA protein levels, suggesting successful repression of *rcoA* (Fig. 6C). We then proceeded to validate the physical interaction between CreA::GFP and TET off-RcoA::3×HA by growing the strains under the same conditions as were used during MS analysis. An interaction between CreA and RcoA was observed in the presence of xylan (CC-derepressing condition) and glucose (CCR), with the latter interaction being stronger (Fig. 6D). These results are therefore in good agreement with the MS data (Data Set S2) and support the idea of a physical interaction between CreA and RcoA under all tested conditions.

Next, we determined whether RcoA is potentially involved in CCR through investigating the expression of the CreA-regulated xylanase *xlnA* gene by RT-qPCR in the wild-type and TET off-RcoA::3×HA strains when grown for 24 h in fructose-rich minimal medium (control) and after transfer to glucose-rich, xylose-rich, or glucose-rich and xylose-rich media for 1 h. Tetracycline was added to the control condition of both strains 3 h prior to transfer to minimal medium supplemented with glucose and/or xylose and maintained thereafter (as a control, the same experiment was carried out in the absence of tetracycline). Repression of *rcoA* had no significant effect on *xlnA* expression under all tested conditions, although a slight reduction in the simultaneous presence of glucose and xylose and tetracycline was observed (Fig. 6E). These results are in agreement with the findings by Hicks et al. (53), who reported a minor role of RcoA in CCR.

## DISCUSSION

Second-generation biofuel production (2G), which aims at converting the polysaccharides stored within plant biomass to biofuels, has gained considerable interest as an alternative to petroleum-based energy sources (56). In order to make 2G biofuel production a cost-effective process on a large scale, bottlenecks such as those limiting increases in and optimization of fungal hydrolytic enzyme production, required for plant biomass deconstruction and production of simple sugars, need to be overcome (57–60). One of the drawbacks in fungal enzyme secretion is the repression of the respective genes in the presence of rapidly metabolizable sugars such as glucose. This phenomenon is known as carbon catabolite repression (CCR) and prevents the utilization of complex carbon sources, thereby allowing the fungus to select the energetically most favorable carbon source (61, 62). In the reference organism A. nidulans, CCR is mediated by the transcriptional regulator CreA- and CreA-mediated repression of target genes and has been thoroughly investigated (63–65). For the present study, we set out to identify and characterize the potential F-box proteins involved in glucose utilization and CCR with the aim of identifying new regulatory mechanisms which could prove useful for biotechnological applications.

An F-box deletion library was generated in A. nidulans and screened for strains that presented increased sensitivity and/or resistance to the glucose analogue 2DG (2-deoxyglucose) and to allyl alcohol (AA). Two F-box proteins, Fbx23 and Fbx47, were identified as potentially being involved in CCR. The Δ*fbx23* strain presented defects in carbon catabolite (CC) derepression, whereas the *Δfbx47* strain had defects in CC repression. In agreement, the expression of *xlnA*, a known CreA target (35,66–68), and the levels of extracellular xylanase and protein secretion were significantly reduced in the Δ*fbx23* strain and increased in the Δ*fbx47* strain under derepressing conditions using xylose as a carbon source. RT-qPCR analysis of *xlnA* in the presence of both glucose and xylose suggests that Fbx23 is directly involved in the transcriptional derepression of *xlnA* by CreA, while Fbx47 seems to be more important for the increased *xlnA* induction in the presence of xylose. To further describe the function of these two proteins and CreA in glucose utilization, protein interaction studies of the TAP-tagged proteins were carried out. Fbx47 could not be tagged at the C terminus, which may have been due to it being highly unstable. It is possible that Fbx47 works independently of an SCF complex, as was shown for the S. cerevisiae F-box protein Rcy1p (69, 70). Further studies are required to investigate the role played by Fbx47 in CCR and glucose utilization, although it is possible that this protein may be involved in CreA nuclear transport, as deletion of *fbx47* resulted in aberrant CreA::GFP cellular localization (Table 1) but did not affect CreA stability (Fig. 4). Alternatively, Fbx47-mediated CCR regulation is even more complex, as the results of RT-qPCR analysis of *xlnA* in the Δ*fbx47* strain were similar to the wild-type strain results seen under CC-repressing conditions (Fig. 2A and B). Further experiments are required to fully understand the function of Fbx47 in CCR.

Mass spectrometry (MS) of interacting Fbx23 and CreA proteins indicated that Fbx23 is part of an SCF ubiquitin ligase complex in the presence of xylan (CC-derepressing condition), whereas CreA forms a repressor complex together with SsnF and RcoA under all tested conditions, suggesting that both transcriptional regulators are also required for normal function of CreA. The physical interaction between CreA and RcoA was confirmed under all conditions, and in agreement with reference 53, RcoA was shown to potentially not be involved in CCR. Alternatively, RcoA could serve as a nonessential scaffold protein or be involved in the recruitment of additional factors that work on target gene chromatin structure via interaction with histones H3 and H4, as previously described (52, 53). Indeed, the addition of glucose and subsequent localization to the nucleus of the CreA repressor complex promote the recruitment of Sin3 (AN1546), which is predicted to form part of a histone deacetylase (HDAC) complex, thereby supporting a role for the CreA repressor complex in target gene chromatin modification.

The Fbx23 SCF ubiquitin ligase complex is predicted to interact with the CreA repressor complex through protein kinase GskA under derepressing conditions, although we were not able to directly prove this by Western blotting of Co-IP analyses due to GskA interacting with different IP resins, resulting in false-positive controls. This indicates that the interaction of the CreA repressor and Fbx23 ubiquitin ligase complexes with GskA is very weak and/or unstable, likely due to subsequent degradation, and can therefore be detected only by highly sensitive methods such as MS. In agreement, MS of IP GskA::3×HA identified potential interactions with RcoA and CkiA, confirming a bridging role for this protein kinase under CC-derepressing conditions. These interactions were lost upon the addition of glucose and were accompanied by GskA::GFP leaving the nucleus, providing additional evidence that the CreA repressor complex is connected to the SCF ubiquitin ligase complex via GskA in order to promote its degradation under CC-derepressing conditions. In agreement, CreA translocates to the nucleus in the presence of glucose (39), and in S. cerevisiae, the CCR Mig1p was shown to physically interact with Rim11p, the homologue of GskA (71).

In the presence of xylan, the Fbx23 and CreA proteins showed patterns of increased degradation, whereas the addition of glucose restored the full-length proteins. This suggests that the CreA repressor complex is ubiquitylated in the presence of xylan, probably resulting in CreA degradation or alteration of function. In agreement, the MS data showed that a ubiquitin precursor molecule is bound to the CreA repressor complex in the presence of xylan and is lost after 15 min under glucose-rich conditions. Furthermore, deletion of *fbx23* resulted in abundant and stable CreA protein which localized to the nucleus in the presence of CC-derepressing conditions, confirming that Fbx23 is required for the destabilization and/or degradation of CreA. This is in agreement with a previous study where increased ubiquitylation of CreA (or of a protein immunoprecipitating together with CreA) was observed in the presence of xylan compared to glucose-rich conditions (27). The deubiquitylation complex CreB/CreC is thought to remove ubiquitin molecules from CreA, thereby leading to the presence of an active CreA protein (38), although no direct interaction of CreA with CreB/CreC was found in this work or in a previous study (72, 73). It therefore remains to be determined with which factor of the repressor complex (de)ubiquitylation takes place.

Upon the addition of glucose to the xylan-grown cultures, the Fbx23 SCF ubiquitin ligase complex is destabilized and/or deactivated via the dissociation of the E3 ligase RbxA, thereby avoiding degradation of the CreA repressor complex and allowing translocation to the nucleus. Nuclear import of CreA could be catalyzed via the karyopherins KapB and KapI, although further studies are required to confirm this. The genome of A. nidulans encodes 14 karyopherins (KapA to KapN), among which 5 (KapA, KapB, KapE, KapF, and KapK) are essential (34). Deletion of the 9 nonessential karyopherin-encoding genes did not result in aberrant CreA::GFP cellular localization, probably due to a redundancy between these receptors (27). Fbx23::GFP was shown to reside within the cytoplasm under CC-repressing and CC-derepressing conditions, with a weaker signal of fluorescence present under derepressing conditions (see Fig. S3 in the supplemental material) due to complex degradation under those conditions (Fig. 4A; see also Fig. S5B).

The MS data of the Fbx23::TAP immunoprecipitation identified casein kinase as a potential interacting partner, being predicted *in silico* to phosphorylate CreA (predicted by NetPhos 3.1; http://www.cbs.dtu.dk/services/NetPhos/). Alternatively, casein kinase may also carry out different functions when interacting with the SCF E3 ligase complex, such as the phosphorylation event that takes place in mammalian cells, where casein kinase I- and II-mediated phosphorylation of target substrates enables subsequent phosphorylation by GSK-3 (homologue of A. nidulans GskA) (74–77). This is an intriguing possibility, as the GskA MS data showed a potential interaction between GskA and CkiA under CC-derepressing conditions. We are currently investigating GskA- and CkiA-targeted CreA phosphorylation sites. Nevertheless, CkiA appears to be involved in CCR, as mutations within the *ckiA* open reading frame or placing CkiA under the control of a thiamine-repressible promoter resulted in increased resistance and sensitivity to 2DG and AA, respectively, indicating that this strain is CC derepressed (Fig. 5).

**FIG 7:**
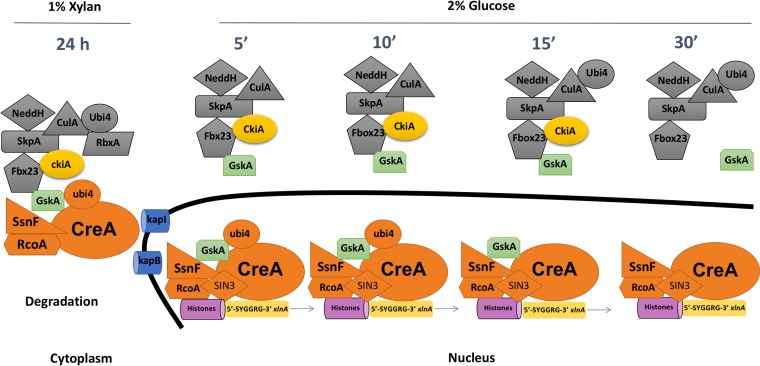
Mechanism of CCR regulation under CC-derepressing (xylan) and CC-repressing (glucose) conditions. In the presence of xylan, CreA forms a protein complex (orange) with RcoA, SsnF, and Ubi4 and is connected via GskA (green) to an SCF ubiquitin ligase complex (gray) and CkiA protein kinase (yellow). This protein complex formation is predicted to promote CreA degradation. Upon the addition of glucose, the CreA repressor complex dissociates from the SCF ubiquitin ligase complex and is imported into the nucleus by the karyopherins KapI and KapB. Inside the nucleus, binding of the CreA repressor complex to target gene promoters such as *xlnA* (5′-SYGGRG-3′) occurs, with RcoA predicted to act on the local chromatin structure (pink). Furthermore, Ubi4 and GskA binding is lost, whereas the complex recruits Sin3, a transcription factor that is involved in chromatin modification. The SCF ubiquitin ligase complex remains in the cytoplasm, where its interaction with GskA and CkiA is also lost after 30 min of glucose treatment.

Based on the aforementioned results, a mechanism regarding the regulation of CreA in the presence of derepressing (xylan) and repressing (glucose) conditions can be depicted (Fig. 7). In the presence of xylan, Fbx23 is functioning within an E3 ligase SCF complex and is connected to CreA via protein kinase GskA, which, together with CkiA, promotes CreA repressor complex phosphorylation and subsequent degradation via ubiquitylation, as previously proposed (27, 38). CreA is localized in the cytoplasm under derepressing conditions, where it forms a complex with SsnF and RcoA and is also marked by the ubiquitin precursor molecule Ubi4. Upon the addition of glucose, the E3 ligase SCF complex dissociates from the CreA repressor complex and CkiA, allowing it to be transported into the nucleus, probably via KapI and KapB karyopherins, and GskA leaves the nucleus. Within the nucleus, the CreA repressor complex loses its interaction with the Ubi4 ubiquitin precursor molecule and protein kinase GskA, whereas interaction with the Sin3 deacetylase complex is enabled, promoting target gene repression (see Data Sets S1 and S2 in the supplemental material).

In summary, this work further contributes to our understanding and provides detailed mechanistic insights of the regulation of CreA-mediated CCR in the reference organism A. nidulans. CreA is likely to be subjected to various posttranslational modifications that occur within a complex regulatory network, involving a multitude of proteins. This work provides a solid basis and novel targets for further investigations and/or genetic manipulations in carbon utilization in biotechnologically relevant fungi with the aim of consolidating 2G biofuel production from plant biomass.

## MATERIALS AND METHODS

### Strain construction and culture medium.

All strains used in this study are listed in Table S1 in the supplemental material, and the primers used are listed in Table S2. The Fbx deletion library was constructed using strain AGB551 (78) as a background strain, by replacing the respective gene with the Aspergillus fumigatus *pyrG* (*AfpyrG*) marker gene. Similarly, strain AGB551 was used to construct all strains which were used for immunoprecipitation assays, where the gene of interest was followed by the respective tag (GFP, TAP, 3×HA) at the 3′ end of the gene, followed by the *AfpyrG* or *AfpyroA* marker gene. All DNA fragments were amplified by PCR (*AfpyrG* was amplified from plasmid pOB435, and *AfpyroA*^*+*^ was amplified from plasmid pOB434) and ligated to each other and into plasmid pUC19 (linearized with SmaI) using an In-Fusion HD cloning kit according to the instructions of the manufacturer (Clontech catalog no. 638911). Vectors harboring the cassettes were then cloned into Escherichia coli, and positive candidates were confirmed by colony PCR. Full cassettes were amplified by PCR from extracted bacterial plasmid DNA (Qiagen Plasmid miniprep) and used for transformation in Aspergillus nidulans as previously described (79). A. nidulans strains harboring the deletion or tagged construct were confirmed by Southern blotting or PCR for homologue integration. All PCRs were carried out using either Q5 or Phusion High Fidelity DNA polymerase (New England Biolabs), according to the manufacturer’s instructions.

10.1128/mBio.00840-18.7 TABLE S2  List of primers used in this work. Download TABLE S2, DOCX file, 0.03 MB. Copyright © 2018 de Assis et al. 2018 de Assis et al. This content is distributed under the terms of the Creative Commons Attribution 4.0 International license.

Strains were grown at 37°C in minimal medium as described previously (80) unless otherwise specified. All liquid-culture-grown mycelia were harvested by vacuum filtration and immediately frozen in liquid nitrogen.

### 2DG screening assay using alamarBlue.

Strains were grown in duplicate from a total of 10^4^ spores for 48 h in a 96-well plate in a total volume of 200 µl minimal medium (MM) per well. MM was supplemented with either xylose or glucose, the specified concentration of 2-deoxy-glucose (2DG), and 10% (vol/vol) alamarBlue (Invitrogen DAL1100). The absorbance at 570 nm and 600 nm of 50 µl of the grown culture supernatant was read using a SpectraMax I3 platform (Molecular Devices). Growth curves are based on the calculated 570-nm/600-nm ratio and subtraction from the negative controls (containing no spores). Positive controls were processed without 2DG and presented 100% of growth of the respective strains.

### Fluorescence microscopy.

Strains were grown in 3 ml MM supplemented with 1% (wt/vol) glucose or xylan in a small petri dish with a coverslip and incubated for 16 h at 22°C instead 37°C due a reduced fluorescence signal (the stability of the GFP tag is increased at lower temperatures). Coverslips containing the fungal germlings were subsequently washed with phosphate-buffered saline (PBS; 140 mM NaCl, 2 mM KCl, 10 mM NaHPO_4_, 1.8 mM KH_2_PO_4_, pH 7.4), and nuclei were stained with 100 ng/ml Hoechst 33258 (Molecular Probes) for 2 min at room temperature. Hyphae were washed again with water and examined using a Zeiss epifluorescence microscope with excitation wavelengths of 359 and 498 nm and emission wavelengths of 461 and 516 nm for Hoechst staining and GFP, respectively. Phase-contrast bright-field and fluorescent images were captured with an AxioCam camera (Carl Zeiss, Inc.) and processed using AxioVision software version 3.1.

### Xylanase assay.

Xylanase (endo-1,4-β-xylanase) assay was performed using Birchwood azo-Xylan (Megazyme International, Bray, Ireland) as a substrate, according to the manufacturer’s instructions.

### Protein extraction and Western blotting.

Crude protein extracts from mycelia were obtained by extraction from ground mycelia with B250 buffer (250 mM NaCl, 100 mM Tris-HCl [pH 7.5], 10% glycerol, 1 mM EDTA, 0.1% NP-40) supplemented with 1.5 ml/liter of 1 M dithiothreitol (DTT), 1 pill/10 ml of (EDTA-free) Complete Mini protease inhibitor cocktail (Roche), 3 ml/liter of 0.5 M benzamidine, 10 ml/liter of 100× phosphatase inhibitors (10 M NaF, 5 M sodium vanadate, 8 M β-glycerol phosphate), and 10 ml/liter of 100 mM phenylmethylsulfonyl fluoride (PMSF). Western blotting was carried out as described previously (80).

### TAP tag protein purification.

Tandem affinity purification (TAP) was performed as described previously (81).

### LC-MS/MS protein identification.

Liquid chromatography (LC)-tandem mass spectrometry (MS/MS) of immunoprecipitated, purified, trypsin-digested, and desalinized samples was carried out as described previously (81).

### Coimmunoprecipitation (Co-IP).

Strains were grown in MM supplemented with 1% xylan for 24 h at 37°C before glucose was added to reach a final concentration of 2% (wt/vol) for 30 min. Protein extraction was carried out as described above. Six milligrams of total protein extract was incubated with 20 µl of Dynabeads protein A (Life Technologies, Inc.; catalog no. 10001D) loaded with 5 µg monoclonal anti-HA antibody (Sigma catalog no. H3663-200UL) for 2 h on a rotator shaker at 4°C. Beads were precipitated on a magnetic rack and washed two times with buffer B250 (see above) and one time with protein extraction buffer (see above). Dissociation of the proteins from the beads was triggered through the addition of SDS-sample buffer and incubation at 95°C for 5 min. Samples were run on a 12% SDS-PAGE gel, and Western blot membranes (see above) were probed for specific antibodies.

### Secretome.

The secretome experiments were carried out as described in reference 80.
