# Supplementary material for: Regulation of Aspergillus nidulans CreA-Mediated Catabolite Repression by the F-Box Proteins Fbx23 and Fbx47
Source: mBio. 2018 Jun 19;9(3):e00840-18. doi: 10.1128/mBio.00840-18 (PMC6016232; doi:10.1128/mBio.00840-18)
Supplement: TABLE S1 [file mbo003183942st1.docx]

**Table S1.** *A. nidulans* strains used in this study

| **Strain** | **Genotype** | **Ref.** |
| --- | --- | --- |
| TNO2a3 | *pyroA4; pyrG89; chaA1; ΔnkuA::argB^+^* | (1) |
| AGB551 | *ΔnkuA::argB; pyrG89; pyroA4; veA^+^* | (2) |
| Fbx23::GFP | *ΔnkuA::argB; pyrG89; pyroA4;* Fbx23-GFP *pyroA4^+^; veA^+^* | This study |
| *∆fbx23::*Fbx23-GFP | *ΔnkuA::argB; Δfbx23::pyrG^+^; pyroA4;* Fbxo23-GFP *pyroA4^+^; veA^+^* | This study |
| Fbx47::GFP | *ΔnkuA::argB; pyrG89; pyroA4;* Fbx47-GFP *pyroA4^+^; veA^+^* | This study |
| *∆fbx47::*Fbx47-GFP | *ΔnkuA::argB; Δfbx47::pyrG^+^; pyroA4;* Fbxo47-GFP *pyroA4^+^; veA^+^* | This study |
| CreA::TAP | *ΔnkuA::argB; pyrG89; pyroA4;* CreA::TAP *pyroA4^+^; veA^+^* | This study |
| CreA::GFP | *ΔnkuA::argB; pyrG89; pyroA4;* CreA::GFP *pyroA4^+^; veA^+^* | This study |
| Fbx23::TAP | *ΔnkuA::argB; pyrG89; pyroA4;* Fbx23::TAP *pyroA4^+^; veA^+^* | This study |
| Fbx23::GFP | *ΔnkuA::argB; pyrG89; pyroA4;* Fbx23::GFP *pyroA4^+^; veA^+^* | This study |
| Fbx47::TAP | *ΔnkuA::argB; pyrG89; pyroA4;* Fbx47(1-1.125)::TAP *pyroA4^+^; veA^+^* | This study |
| Fbx47.1::TAP | *ΔnkuA::argB; pyrG89; pyroA4;* Fbx47(1-792)::TAP *pyroA4^+^; veA^+^* | This study |
| Fbx47.2::TAP | *ΔnkuA::argB; pyrG89; pyroA4;* Fbx47(1-780)::TAP *pyroA4^+^; veA^+^* | This study |
| Fbx47::GFP | *ΔnkuA::argB; pyrG89; pyroA4;* Fbx47::GFP *pyroA4^+^; veA^+^* | This study |
| *∆ckiB* | *pyrG89; wA3; argB2; ∆nkuAku70::argB; pyroA4; sE15 nirA14 chaA1 fwA1; ∆ckiB::pyrG^+^* | (3) |
| *∆gskA* | *pyrG89; wA3; argB2; ∆nkuAku70::argB; pyroA4; sE15 nirA14 chaA1 fwA1; ∆gskA::pyrG^+^* | (3) |
| *∆gskA* CreA::GFP | *pyrG89; wA3; argB2; ∆nkuAku70::argB; pyroA4; sE15 nirA14 chaA1 fwA1; ∆gskA::pyrG^+^;* CreA::GFP *pyroA4^+^* | This study |
| *∆fbx23* CreA::GFP | *ΔnkuA::argB; pyroA4; Δfbx23::pyrG^+^;* CreA::GFP *pyroA4^+^; veA^+^* | This study |
| *∆fbx47* CreA::GFP | *ΔnkuA::argB; pyroA4; Δfbx47::pyrG^+^;* CreA::GFP *pyroA4^+^; veA^+^* | This study |
| *∆ckiB* CreA::GFP | *pyrG89; wA3; argB2; ∆nkuAku70::argB; pyroA4; sE15 nirA14 chaA1 fwA1; ∆ckiB::pyrG^+^;* CreA::GFP *pyroA4^+^* | This study |
| GskA::3xHA | *ΔnkuA::argB; pyrG89; pyroA4;* GskA::3xHA *pyrG^+^; veA^+^* | This study |
| GskA::3xHA CreA::GFP | *ΔnkuA::argB; pyrG89; pyroA4;* GskA::3xHA *pyrG^+^;* CreA::GFP *pyroA4^+^; veA^+^* | This study |
| GskA::3xHA Fbx23::GFP | *ΔnkuA::argB; pyrG89; pyroA4;* GskA::3xHA *pyrG^+^;* Fbx23::GFP *pyroA4^+^; veA^+^* | This study |
| Fbx23::3xHA | *ΔnkuA::argB; pyroA4;* Fbx23::3xHA *pyrG ^+^; veA^+^* | This study |
| Fbx23::3xHA  GskA::GFP (sick) | *ΔnkuA::argB;* Fbx23::3xHA *pyrG ^+^;* GskA::GFP *pyroA4^+^; veA^+^* | This study |
| GskA::GFP | *ΔnkuA::argB; pyrG89;* GskA::GFP *pyroA4 ^+^; veA^+^* | This study |
| GskA::GFP  CreA::FLAG | *ΔnkuA::argB; chaA1;* CreA::FLAG *pyrG^+^;* GskA::GFP *pyroA4^+^* | This study |
| CreA::FLAG | *ΔnkuA::argB; chaA1; pyroA4;* CreA::FLAG *pyrG^+^* | This study |
| ATH40 | *pabaA1* | (4) |
| CkiA::GFP (VIE172) | CkiA::GFP *pyrG*^+^; *pabaB22*; *riboB2* | (4) |
| ckiA2 (VIE174) | ckiA2; *pyroA4*; yA2 | (4) |
| ckiA1919 (VIE179) | *pyrG89; pabaA1* | (4) |
| ckiA102 (CS1947) | *pabaA1;* yA2 | (4) |
| BF110 | *biA1 CreA^d30^* | (5) |
| CreA::GFP RcoA::3xHA TET off | *ΔnkuA::argB; CreA::GFP pyroA4^+^; TET Off RcoA::3xHA pyrG^+^; ^veA+^* | This study |
| *∆fbx1* | *ΔnkuA::argB; pyrG89; pyroA4; veA^+^  ∆AN6086::pyrG^+^* | This study |
| *∆fbx2* | *ΔnkuA::argB; pyrG89; pyroA4; veA^+^  ∆AN6816::pyrG^+^* | This study |
| *∆fbx3* | *ΔnkuA::argB; pyrG89; pyroA4; veA^+^  ∆AN6183::pyrG^+^* | This study |
| *∆fbx4* | *ΔnkuA::argB; pyrG89; pyroA4; veA^+^  ∆AN6552::pyrG^+^* | This study |
| *∆fbx5* | *ΔnkuA::argB; pyrG89; pyroA4; veA^+^  ∆AN7956::pyrG^+^* | This study |
| *∆fbx6* | *ΔnkuA::argB; pyrG89; pyroA4; veA^+^  ∆AN9187::pyrG^+^* | This study |
| *∆fbx7* | *ΔnkuA::argB; pyrG89; pyroA4; veA^+^  ∆AN0249::pyrG^+^* | This study |
| *∆fbx8* | *ΔnkuA::argB; pyrG89; pyroA4; veA^+^  ∆AN0353::pyrG^+^* | This study |
| *∆fbx9* | *ΔnkuA::argB; pyrG89; pyroA4; veA^+^  ∆AN10061::pyrG^+^* | This study |
| *∆fbx10* | *ΔnkuA::argB; pyrG89; pyroA4; veA^+^  ∆AN0460::pyrG^+^* | This study |
| *∆fbx11* | *ΔnkuA::argB; pyrG89; pyroA4; veA^+^  ∆AN0557::pyrG^+^* | This study |
| *∆fbx12* | *ΔnkuA::argB; pyrG89; pyroA4; veA^+^  ∆AN2029::pyrG^+^* | This study |
| *∆fbx13* | *ΔnkuA::argB; pyrG89; pyroA4; veA^+^  ∆AN2348::pyrG^+^* | This study |
| *∆fbx14* | *ΔnkuA::argB; pyrG89; pyroA4; veA^+^  ∆AN2353::pyrG^+^* | This study |
| *∆fbx15* | *ΔnkuA::argB; pyrG89; pyroA4; veA^+^  ∆AN2505::pyrG^+^* | This study |
| *∆fbx16* | *ΔnkuA::argB; pyrG89; pyroA4; veA^+^  ∆AN2636::pyrG^+^* | This study |
| *∆fbx17* | *ΔnkuA::argB; pyrG89; pyroA4; veA^+^  ∆AN2861::pyrG^+^* | This study |
| *∆fbx18* | *ΔnkuA::argB; pyrG89; pyroA4; veA^+^  ∆AN4488::pyrG^+^* | This study |
| *∆fbx19* | *ΔnkuA::argB; pyrG89; pyroA4; veA^+^  ∆AN4510::pyrG^+^* | This study |
| *∆fbx20* | *ΔnkuA::argB; pyrG89; pyroA4; veA^+^  ∆AN4535::pyrG^+^* | This study |
| *∆fbx21* | *ΔnkuA::argB; pyrG89; pyroA4; veA^+^  ∆AN5509::pyrG^+^* | This study |
| *∆fbx22* | *ΔnkuA::argB; pyrG89; pyroA4; veA^+^  ∆AN5517::pyrG^+^* | This study |
| *∆fbx23* | *ΔnkuA::argB; pyrG89; pyroA4; veA^+^  ∆AN5593::pyrG^+^* | This study |
| *∆fbx24* | *ΔnkuA::argB; pyrG89; pyroA4; veA^+^  ∆AN6217::pyrG^+^* | This study |
| *∆fbx25* | *ΔnkuA::argB; pyrG89; pyroA4; veA^+^  ∆AN6359::pyrG^+^* | This study |
| *∆fbx26* | *ΔnkuA::argB; pyrG89; pyroA4; veA^+^  ∆AN7086::pyrG^+^* | This study |
| *∆fbx27* | *ΔnkuA::argB; pyrG89; pyroA4; veA^+^  ∆AN9113::pyrG^+^* | This study |
| *∆fbx28* | *ΔnkuA::argB; pyrG89; pyroA4; veA^+^  ∆AN5209::pyrG^+^* | This study |
| *∆fbx29* | *ΔnkuA::argB; pyrG89; pyroA4; veA^+^  ∆AN2933::pyrG^+^* | This study |
| *∆fbx30* | *ΔnkuA::argB; pyrG89; pyroA4; veA^+^  ∆AN9059::pyrG^+^* | This study |
| *∆fbx31* | *ΔnkuA::argB; pyrG89; pyroA4; veA^+^  ∆AN0307::pyrG^+^* | This study |
| *∆fbx32* | *ΔnkuA::argB; pyrG89; pyroA4; veA^+^  ∆AN1693::pyrG^+^* | This study |
| *∆fbx33* | *ΔnkuA::argB; pyrG89; pyroA4; veA^+^  ∆AN2106::pyrG^+^* | This study |
| *∆fbx34* | *ΔnkuA::argB; pyrG89; pyroA4; veA^+^  ∆AN2364::pyrG^+^* | This study |
| *∆fbx35* | *ΔnkuA::argB; pyrG89; pyroA4; veA^+^  ∆AN2806::pyrG^+^* | This study |
| *∆fbx36* | *ΔnkuA::argB; pyrG89; pyroA4; veA^+^  ∆AN3203::pyrG^+^* | This study |
| *∆fbx37* | *ΔnkuA::argB; pyrG89; pyroA4; veA^+^  ∆AN4237::pyrG^+^* | This study |
| *∆fbx38* | *ΔnkuA::argB; pyrG89; pyroA4; veA^+^  ∆AN5075::pyrG^+^* | This study |
| *∆fbx39* | *ΔnkuA::argB; pyrG89; pyroA4; veA^+^  ∆AN5161::pyrG^+^* | This study |
| *∆fbx40* | *ΔnkuA::argB; pyrG89; pyroA4; veA^+^  ∆AN5568::pyrG^+^* | This study |
| *∆fbx41* | *ΔnkuA::argB; pyrG89; pyroA4; veA^+^  ∆AN5933::pyrG^+^* | This study |
| *∆fbx42* | *ΔnkuA::argB; pyrG89; pyroA4; veA^+^  ∆AN6777::pyrG^+^* | This study |
| *∆fbx43* | *ΔnkuA::argB; pyrG89; pyroA4; veA^+^  ∆AN6999::pyrG^+^* | This study |
| *∆fbx44* | *ΔnkuA::argB; pyrG89; pyroA4; veA^+^  ∆AN8008::pyrG^+^* | This study |
| *∆fbx45* | *ΔnkuA::argB; pyrG89; pyroA4; veA^+^  ∆AN8098::pyrG^+^* | This study |
| *∆fbx46* | *ΔnkuA::argB; pyrG89; pyroA4; veA^+^  ∆AN8776::pyrG^+^* | This study |
| *∆fbx47* | *ΔnkuA::argB; pyrG89; pyroA4; veA^+^  ∆AN8909::pyrG^+^* | This study |
| *∆fbx48* | *ΔnkuA::argB; pyrG89; pyroA4; veA^+^  ∆AN10073::pyrG^+^* | This study |
| *∆fbx49* | *ΔnkuA::argB; pyrG89; pyroA4; veA^+^  ∆AN10117::pyrG^+^* | This study |
| *∆fbx50* | *ΔnkuA::argB; pyrG89; pyroA4; veA^+^  ∆AN10516::pyrG^+^* | This study |
| *∆fbx51* | *ΔnkuA::argB; pyrG89; pyroA4; veA^+^  ∆AN8462::pyrG^+^* | This study |
| *∆fbx52* | *ΔnkuA::argB; pyrG89; pyroA4; veA^+^  ∆AN2837::pyrG^+^* | This study |
| *∆fbx53* | *ΔnkuA::argB; pyrG89; pyroA4; veA^+^  ∆AN5263::pyrG^+^* | This study |
| *∆fbx54* | *ΔnkuA::argB; pyrG89; pyroA4; veA^+^  ∆AN1705::pyrG^+^* | This study |
| *∆fbx55* | *ΔnkuA::argB; pyrG89; pyroA4; veA^+^  ∆AN5941::pyrG^+^* | This study |
| *∆fbx56* | *ΔnkuA::argB; pyrG89; pyroA4; veA^+^  ∆AN6152::pyrG^+^* | This study |
| *∆fbx57* | *ΔnkuA::argB; pyrG89; pyroA4; veA^+^  ∆AN6944::pyrG^+^* | This study |
| *∆fbx58* | *ΔnkuA::argB; pyrG89; pyroA4; veA^+^  ∆AN8051::pyrG^+^* | This study |
| *∆fbx59* | *ΔnkuA::argB; pyrG89; pyroA4; veA^+^  ∆AN10347::pyrG^+^* | This study |
| *∆fbx60* | *ΔnkuA::argB; pyrG89; pyroA4; veA^+^  ∆AN6887::pyrG^+^* | This study |
| *∆fbx61* | *ΔnkuA::argB; pyrG89; pyroA4; veA^+^  ∆AN7964::pyrG^+^* | This study |
| *∆fbx62* | *ΔnkuA::argB; pyrG89; pyroA4; veA^+^  ∆AN8128::pyrG^+^* | This study |
| *∆fbx63* | *ΔnkuA::argB; pyrG89; pyroA4; veA^+^  ∆AN2540::pyrG^+^* | This study |
| *∆fbx64* | *ΔnkuA::argB; pyrG89; pyroA4; veA^+^  ∆AN2808::pyrG^+^* | This study |
| *∆fbx65* | *ΔnkuA::argB; pyrG89; pyroA4; veA^+^  ∆AN3371::pyrG^+^* | This study |
| *∆fbx66* | *ΔnkuA::argB; pyrG89; pyroA4; veA^+^  ∆AN5034::pyrG^+^* | This study |
| *∆fbx67* | *ΔnkuA::argB; pyrG89; pyroA4; veA^+^  ∆AN5576::pyrG^+^* | This study |
| *∆fbx68* | *ΔnkuA::argB; pyrG89; pyroA4; veA^+^  ∆AN2183::pyrG^+^* | This study |
| *∆fbx69* | *ΔnkuA::argB; pyrG89; pyroA4; veA^+^  ∆AN6634::pyrG^+^* | This study |
| *∆fbx70* | *ΔnkuA::argB; pyrG89; pyroA4; veA^+^  ∆AN4548::pyrG^+^* | This study |
| *∆fbx71* | *ΔnkuA::argB; pyrG89; pyroA4; veA^+^  ∆AN5714::pyrG^+^* | This study |
| *∆fbx72* | *ΔnkuA::argB; pyrG89; pyroA4; veA^+^  ∆AN4149::pyrG^+^* | This study |
| *∆fbx73* | *ΔnkuA::argB; pyrG89; pyroA4; veA^+^  ∆AN6625::pyrG^+^* | This study |
| *∆fbx74* | *ΔnkuA::argB; pyrG89; pyroA4; veA^+^  ∆AN0703::pyrG^+^* | This study |

Reference:

1. Nayak T, Szewczyk E, Oakley CE, Osmani A, Ukil L, Murray SL, Hynes MJ, Osmani SA, Oakley BR. 2006. A versatile and efficient gene-targeting system for Aspergillus nidulans. Genetics 172:1557–1566.

2. Bayram Ö, Bayram ÖS, Ahmed YL, Maruyama J ichi, Valerius O, Rizzoli SO, Ficner R, Irniger S, Braus GH. 2012. The Aspergillus nidulans MAPK module AnSte11-Ste50-Ste7-Fus3 controls development and secondary metabolism. PLoS Genet 8.

3. De Souza CP, Hashmi SB, Osmani AH, Andrews P, Ringelberg CS, Dunlap JC, Osmani SA. 2013. Functional analysis of the Aspergillus nidulans kinome. PLoS One 8:e58008.

4. Apostolaki A, Harispe L, Calcagno-Pizarelli AM, Vangelatos I, Sophianopoulou V, Arst HN, Peñalva MA, Amillis S, Scazzocchio C. 2012. Aspergillus nidulans CkiA is an essential casein kinase I required for delivery of amino acid transporters to the plasma membrane. Mol Microbiol 84:530–49.

5. Arst HN, Tollervey D, Dowzer CEA, Kelly JM. 1990. Notes An inversion truncating the creA gene of Aspergillus niduians results in carbon catabolite derepression. Mol Microbiol 4:851–854.
