# Supplementary figures and images for: Regulation of Aspergillus nidulans CreA-Mediated Catabolite Repression by the F-Box Proteins Fbx23 and Fbx47
Source: mBio. 2018 Jun 19;9(3):e00840-18. doi: 10.1128/mBio.00840-18 (PMC6016232; doi:10.1128/mBio.00840-18)

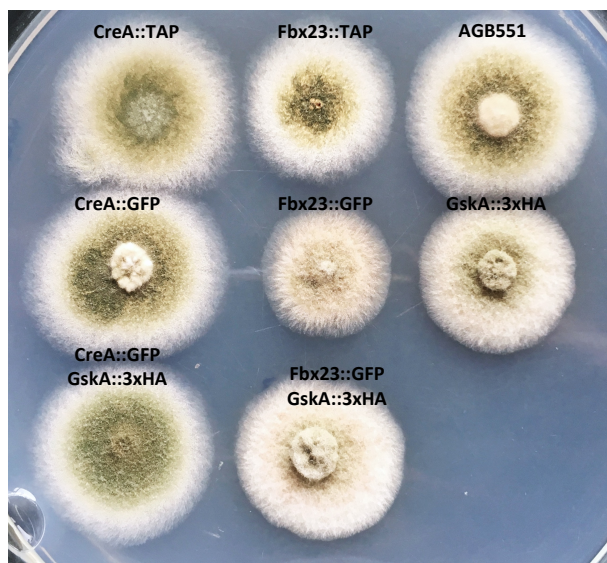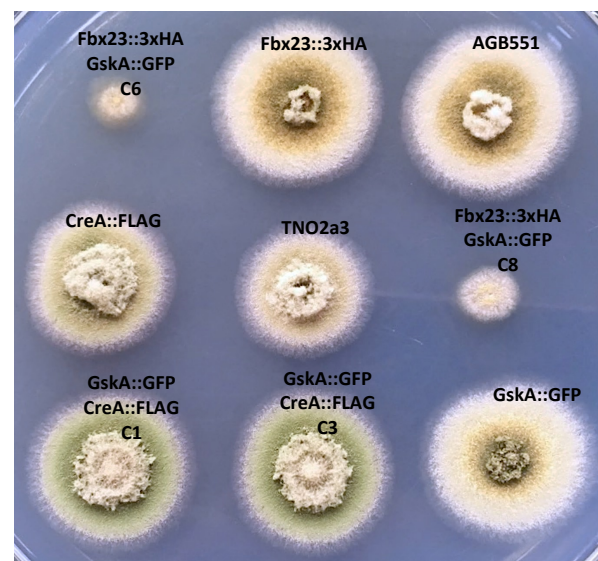

Supplement: FIG S1 [file mbo003183942sf1.pdf]

**A**

*Fbx47-TAP* (Full length 1-1.125 bp)

*Fbx47.1-TAP* (F1 1-792 bp)

*Fbx47.2-TAP* (F2 1-780 bp)

**B**

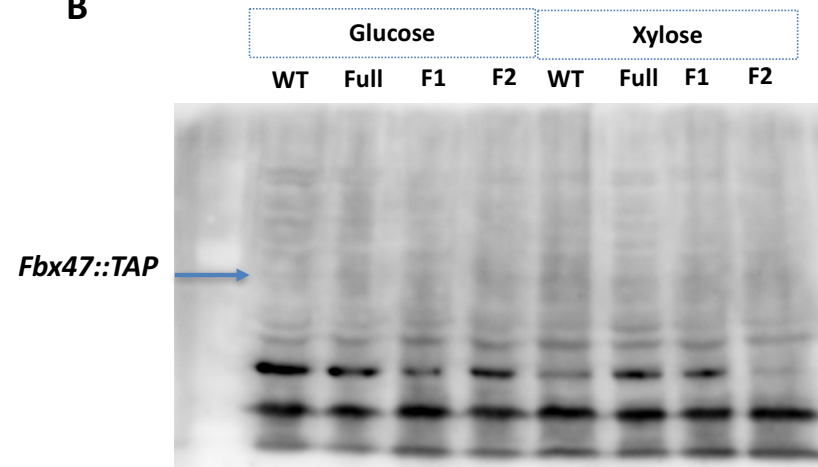

Supplement: FIG S2 [file mbo003183942sf2.pdf]

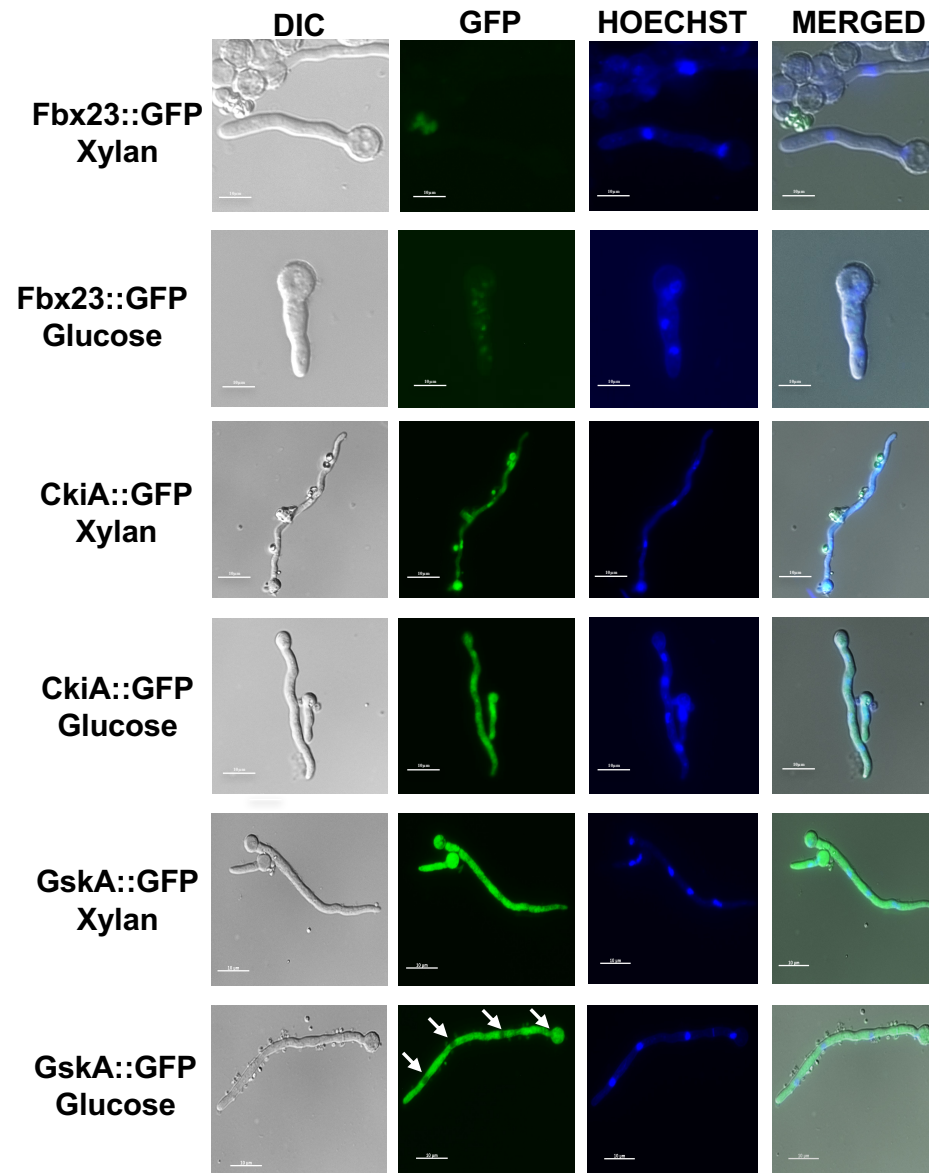

Supplement: FIG S3 [file mbo003183942sf3.pdf]

**A**

IP:HA Western blot: anti-GFP

**Xylan (24 hrs)**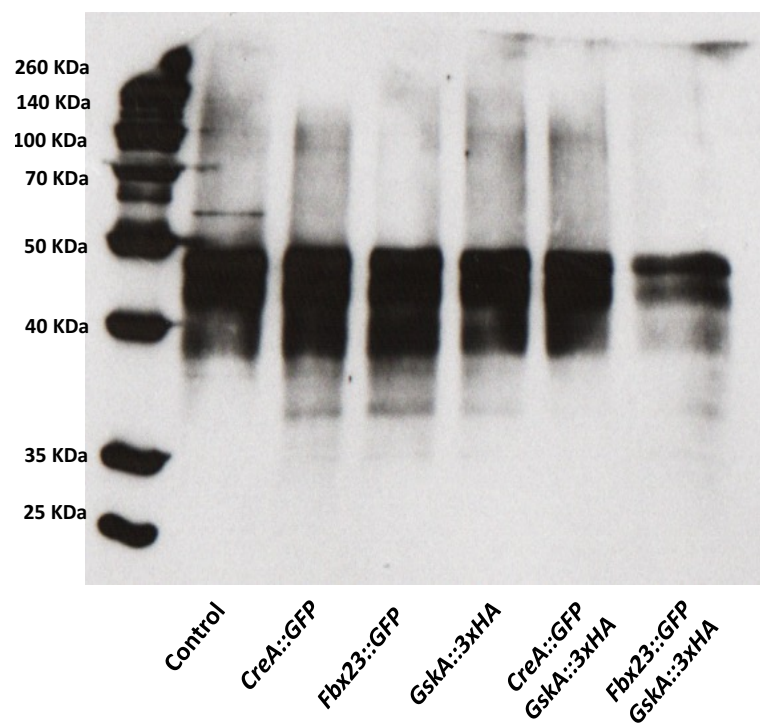**B****Glucose (30 min)**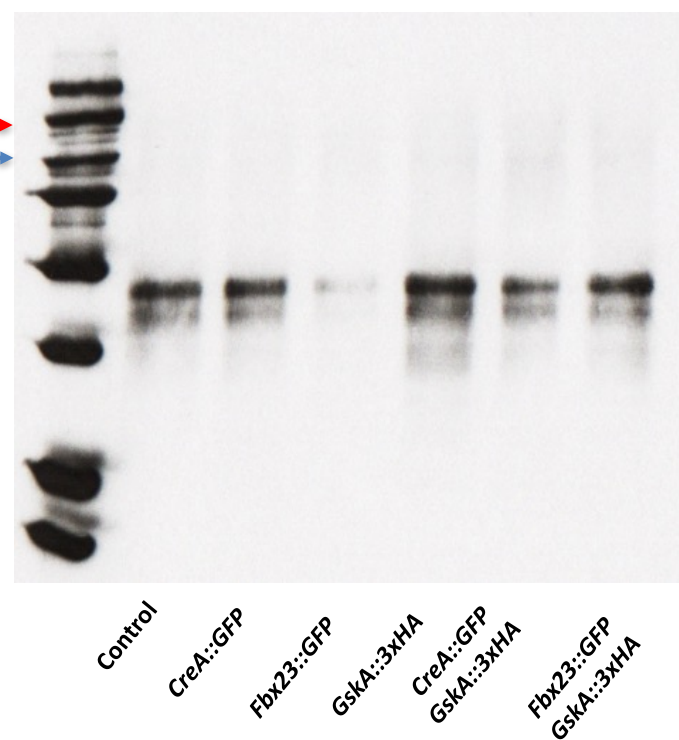**S5**

Supplement: FIG S4 [file mbo003183942sf4.pdf]
