# Supplementary material for: Regulation of Aspergillus nidulans CreA-Mediated Catabolite Repression by the F-Box Proteins Fbx23 and Fbx47
Source: mBio. 2018 Jun 19;9(3):e00840-18. doi: 10.1128/mBio.00840-18 (PMC6016232; doi:10.1128/mBio.00840-18)
Supplement: FIG S5 [file mbo003183942sf5.pdf]

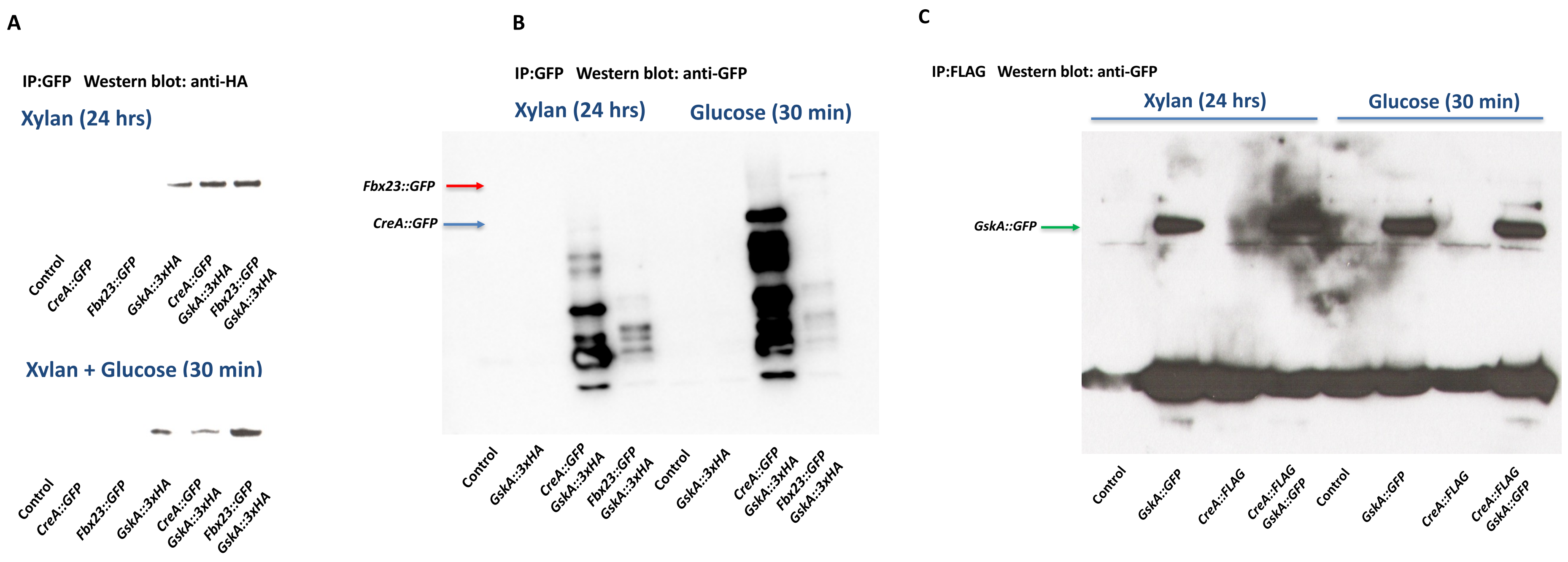
A
IP:GFP Western blot: anti-HA
Xylan (24 hrs)
Control
CreA::GFP
Fbx23::GFP
GskA::3xHA
CreA::GFP
GskA::3xHA
Fbx23::GFP
GskA::3xHA
Xylan + Glucose (30 min)
Control
CreA::GFP
Fbx23::GFP
GskA::3xHA
CreA::GFP
GskA::3xHA
Fbx23::GFP
GskA::3xHA
B
IP:GFP Western blot: anti-GFP
Xylan (24 hrs)
Glucose (30 min)
Fbx23::GFP
CreA::GFP
Control
GskA::3xHA
CreA::GFP
GskA::3xHA
Fbx23::GFP
GskA::3xHA
Control
GskA::3xHA
CreA::GFP
GskA::3xHA
Fbx23::GFP
GskA::3xHA
C
IP:FLAG Western blot: anti-GFP
Xylan (24 hrs)
Glucose (30 min)
GskA::GFP
Control
GskA::GFP
CreA::FLAG
CreA::FLAG
GskA::GFP
Control
GskA::GFP
CreA::FLAG
CreA::FLAG
GskA::GFP
