# Supplementary material for: Regulation of Aspergillus nidulans CreA-Mediated Catabolite Repression by the F-Box Proteins Fbx23 and Fbx47
Source: mBio. 2018 Jun 19;9(3):e00840-18. doi: 10.1128/mBio.00840-18 (PMC6016232; doi:10.1128/mBio.00840-18)
Supplement: TABLE S2 [file mbo003183942st2.docx]

**Table S2. List of primers used in this work.**

| **Primer** | **Sequence** |
| --- | --- |
| OZG916 (PyrG) | GGAGGTGGTAGCGGTGGT |
| OZG694 (PyrG) | CTGTCTGAGAGGAGGCACTGAT |
| LJA1 (*gskA*) | TTCGAGCTCGGTACCCGATCCTGAGCTACAAGTCCAAG |
| LJA2 (*gskA*) | CACCGCTACCACCTCCGTCAAGGTGTGCCATCATTTC |
| LJA3 (*gskA*) | CAGTGCCTCCTCTCAGACAGGTCAACGTCATAGTGCTGAT |
| LJA4 (*gskA*) | CGACTCTAGAGGATCCCCCGTCGAACTTTGACCACTAGAG |
| LJA75.1 (Fbx47.1) | CACCGCTACCACCTCCACAGTCGATCATGCACCCCA |
| LJA75.2 (Fbx47.2) | CACGCTACCACCTCCTGCACCCCATGCAAGCTGAATA |
| MUL55 (*CreA::GFP/TAP*) | TTCGAGCTCGGTACCCGCTCCGATTCCGATAACCCTC |
| MUL56 (*CreA::GFP/TAP*) | CATCCTCCATTCCGGCTCTC |
| MUL59 (*CreA::GFP/TAP*) | CGTCCAGGATACCATATCTCC |
| MUL60 (*CreA::GFP/TAP*) | ACTCTAGAGGATCCCCCATTACTATCCGTGCATCGCG |
| MUL67 (*F23::GFP/TAP*) | TTCGAGCTCGGTACCCGCACATCTGCTTGACCTCCAG |
| MUL68 (*F23::GFP/TAP*) | GTCTCAAGTATGGAGGAGGGC |
| MUL69 (*F23::GFP/TAP*) | CACCGCTACCACCTCCTAACCCCCTGAAAAACTGACAAG |
| MUL70 (*F23::GFP/TAP*) | GCCTCCTCTCAGACAGCATCCGATTTGCTGGGGTGG |
| MUL71 (*F23::GFP/TAP*) | CCAGTTCCAAATCGTAGCGAG |
| MUL72 (*F23::GFP/TAP*) | ACTCTAGAGGATCCCCCGAGGAAGCTAGAACGTATGAC |
| MUL73 (*F47::GFP/TAP*) | TTCGAGCTCGGTACCCCAGCGTGATCGGTCCAACTTG |
| MUL74 (*F47::GFP/TAP*) | CTGGATGTTCCACTGCCATCC |
| MUL75 (*F47::GFP/TAP*) | CACCGCTACCACCTCCAATGAGACGGACAAGGACTAGC |
| MUL76 (*F47::GFP/TAP*) | GCCTCCTCTCAGACAGGATCACTGCAGAGGATAGCATG |
| MUL77 (*F47::GFP/TAP*) | GTTGAATCCGGACGAGATGTC |
| MUL78 (*F47::GFP/TAP*) | ACTCTAGAGGATCCCCGTTGAATCCGGACGAGATGTC |
| pRS426-gskA 5’ UTR F | GTAACGCCAGGGTTTTCCCAGTCACGACGTGAGCTACAAGTCCAAGGGAT |
| gskA-GFP RV | CCATACCACCGCTACCACCTCCGTCAAGGTGTGCCATCATTTC |
| GFP-3’ UTR gskA | ATCAGTGCCTCCTCTCAGACAGGTCAACGTCATAGTGCTGA |
| 3’ UTR gskA-pRS426 | GCGGATAACAATTTCACACAGGAAACAGCCGCCGAGAATCTAACCTGTTT |
| pRS426-fbx23 5’ UTR F | GTAACGCCAGGGTTTTCCCAGTCACGACGGCACATCTGCTTGACCTCCAG |
| fbx23-3xHA RV | ATAACCACCGCTACCACCTCCTAACCCCCTGAAAAACTGACA |
| 3xHA-3’ UTR fbx23 | ATCAGTGCCTCCTCTCAGACAGCATCCGATTTGCTGGGGTG |
| 3’ UTR fbx23-pRS426 | GCGGATAACAATTTCACACAGGAAACAGCCGAGGAAGCTAGAACGTATGAC |
| 3’ UTR gskA-pRS426 | GCGGATAACAATTTCACACAGGAAACAGCCGCCGAGAATCTAACCTGTTT |
| 3X - 5’ UTR RcoA | GATAACCACCGCTACCACCTCCTCAGCGCACCCCTAGGAAACTG |
| pRS426-5’ UTR RcoA | GTAACGCCAGGGTTTTCCCAGTCACGACGATTGAGCGCATGGTTTGC |
| 5’ UTR RcoA - TET off RV | GACAGACCAGCCGAGGGAAGTCAGCGCACCCCTAGGAAAC |
| TET Off - RcoA FW | GCTTGAGCAGACATCACCGATGCGCAGCATTGACCAAC |
| RcoA - 3xHA RV | ATAACCACCGCTACCACCTCCCCGTCCAGTGTACGCGGAGTA |
| 3xHA - 3’ UTR RcoA FW | ATCAGTGCCTCCTCTCAGACAGTAATCATTGAATTTGGGAA |
| 3’ UTR RcoA RV | GGTGGGGAATCATCAATCTT |
| TET Off FW | CTTCCCTCGGCTGGTCTGTC |
| TET Off RV | CGGTGATGTCTGCTCAAGCG |
